# The Receptor Tyrosine Kinase Alk Controls Neurofibromin Functions in Drosophila Growth and Learning

**DOI:** 10.1371/journal.pgen.1002281

**Published:** 2011-09-15

**Authors:** Jean Y. Gouzi, Anastasios Moressis, James A. Walker, Anthi A. Apostolopoulou, Ruth H. Palmer, André Bernards, Efthimios M. C. Skoulakis

**Affiliations:** 1 Institute of Cellular and Developmental Biology, Biomedical Sciences Research Centre "Alexander Fleming," Vari, Greece; 2 Department of Basic Sciences, School of Health Sciences, National and Kapodistrian University of Athens, Athens, Greece; 3 Center for Cancer Research, Massachusetts General Hospital and Harvard Medical School, Charlestown, Massachusetts, United States of America; 4 Department of Molecular Biology, Umeå University, Umeå, Sweden; University of Pennsylvania, United States of America

## Abstract

*Anaplastic Lymphoma Kinase* (*Alk*) is a Receptor Tyrosine Kinase (RTK) activated in several cancers, but with largely unknown physiological functions. We report two unexpected roles for the Drosophila ortholog *dAlk*, in body size determination and associative learning. Remarkably, reducing neuronal dAlk activity increased body size and enhanced associative learning, suggesting that its activation is inhibitory in both processes. Consistently, dAlk activation reduced body size and caused learning deficits resembling phenotypes of null mutations in *dNf1*, the Ras GTPase Activating Protein-encoding conserved ortholog of the Neurofibromatosis type 1 (NF1) disease gene. We show that dAlk and dNf1 co-localize extensively and interact functionally in the nervous system. Importantly, genetic or pharmacological inhibition of dAlk rescued the reduced body size, adult learning deficits, and Extracellular-Regulated-Kinase (ERK) overactivation *dNf1* mutant phenotypes. These results identify dAlk as an upstream activator of dNf1-regulated Ras signaling responsible for several *dNf1* defects, and they implicate human Alk as a potential therapeutic target in NF1.

## Author Summary

Neurofibromatosis-1 (NF1) syndrome is a common (1/3,000 births) genetic disorder affecting multiple organ systems, including the nervous system. Its clinical features include short stature, learning disabilities, and several types of benign and malignant tumors. NF1 is caused by mutations that inactivate the *NF1* gene, a crucial negative regulator of Ras signaling. Although unregulated Ras signaling is a hallmark of NF1, the specific Ras signaling pathways responsible for disease development remain largely unknown. The Drosophila and human *Nf1* genes are highly conserved; and, as in patients, mutant flies are smaller than usual and present deficient learning. Here, we identified the Drosophila Receptor Tyrosine Kinase dAlk as a negative regulator of organismal growth and olfactory learning. We show that excessive dAlk activation results in growth and learning defects similar to those of *Nf1* mutants. Genetic suppression studies and pharmacological inhibition indicate dAlk as a critical upstream activator of Nf1-regulated neuronal Ras/ERK signals that contribute to size determination and learning. Importantly, our results strongly suggest that Alk represents a novel, highly specific, and promising therapeutic target in human NF1.

## Introduction

Receptor Tyrosine Kinases (RTKs) are transmembrane proteins with intrinsic kinase activity directed in part towards tyrosine residues within their own carboxy-terminal tails. They play pivotal roles in most tissues, including the central nervous system (CNS), by transducing extracellular ligand binding events into intracellular signals. A major signaling pathway activated by RTKs is the Ras/ERK (Extracellular signal Regulated Kinase) cascade [1]–[4]. Initially thought to be mostly involved in cell proliferation and differentiation, recent work has increasingly implicated various components and regulators of this signaling cascade in neuronal plasticity and memory formation [4]. However, although most RTKs should, in principle, be able to activate Ras/ERK signaling, only few among the 58 human receptors have been functionally linked to cognitive processes [5]. Even in Drosophila, a system with powerful genetics and resident homologs of most mammalian RTKs [6], evidence implicating these receptors in learning and memory remains scant [5]. The *linotte/derailed* RTK (an ortholog of RYK) is the only Drosophila family member implicated in learning and memory to date [7]. However, deficits in adult neuroplasticity associated with mutations in this gene appear at least partially attributable to abnormal brain development [8]. In addition, *linotte/derailed* is an atypical RTK, devoid of intrinsic kinase activity. Evidence suggesting involvement of at least one typical RTK in olfactory associative learning and memory in the fly comes from work on Drk, an adaptor protein that binds active tyrosine phosphorylated receptors [1], [2]. Reducing Drk levels results in defective olfactory learning and memory [9], suggesting that at least one RTK may be involved in this process.

To identify RTKs potentially involved in Drosophila learning and memory, we determined the family members which are expressed in the adult CNS. The fly ortholog of Anaplastic Lymphoma Kinase (Alk) was among genes showing prominent expression in this screen. Vertebrate Alk, and its dAlk Drosophila ortholog, are members of the insulin receptor subfamily of RTKs, [10], [11]. Two related secreted proteins, pleiotrophin and midkine, can activate vertebrate Alk, although whether they do so directly by interacting with Alk, or indirectly by modulating the activity of a transmembrane tyrosine phosphatase, remains controversial [11]. As for most RTKs, Alk activation results in the recruitment of adaptor proteins, such as IRS-1, Shc and FRS2 and initiation of intracellular signaling pathways, including the canonical Ras/ERK cascade [11], [12]. Aberrant activation of the Alk kinase by chromosomal translocations or point mutations has been causally implicated in anaplastic large cell lymphoma, non-small cell lung cancer, and neuroblastoma [11], [13]–[17]. Alk signaling may also be a rate limiting factor controlling the growth of glioblastoma cells [14] and non-synonymous polymorphisms in the gene may be associated with schizophrenia [18]. While recent reports have generated much excitement about Alk as a therapeutic target in lung cancer [19], the normal roles of vertebrate Alk remain poorly understood [11].

Drosophila dAlk functions in visceral muscle development in the embryo [20]–[22], in axonal targeting in the retina [23] and in synaptic signaling at the larval neuromuscular junction [24]. Although the Drosophila *miple1* and *miple2* genes predict pleiotrophin- and midkine-related proteins, the *bona fide* dAlk-activating ligand is the secreted protein Jelly belly (Jeb) [11]. As reported here, we found dAlk to be widely expressed in the adult brain, but to be especially abundant in the calyces of the mushroom bodies (MBs), neuronal structures essential for olfactory learning and memory [25], where Drk is also preferentially expressed [9]. Prompted by these observations, we investigated whether dAlk functions in associative learning. Our results identify dAlk as the first active RTK involved in olfactory learning, but also in body size determination. Intriguingly, dAlk shares both of these disparate functions with dNf1, the ortholog of the human neurofibromatosis type 1 (NF1) tumor suppressor gene.

NF1 affects 1 in 3,000 individuals worldwide and is the most common genetic disease associated with an increased cancer predisposition. Hallmark NF1 tumors are benign neurofibromas and malignant peripheral nerve sheath tumors. Learning defects in most children with NF1, skin pigmentation abnormalities, and short stature are among other common symptoms of this multi-system disorder. NF1 is caused by loss of function mutations in a gene, termed *NF1*, encoding an evolutionary conserved Ras GTPase Activating Protein (GAP), termed neurofibromin. While excess Ras signaling due to loss of neurofibromin is widely believed to be responsible for most, if not all NF1 defects, no effective therapy for any NF1 defect has yet been devised. Moreover, Ras is a central component of multiple signaling pathways, and which of these specifically contribute to disease development remains only partially understood [26], [27].

Drosophila neurofibromin is approximately 60% identical to the human protein, and *dNf1* null mutants are viable, fertile, and normally patterned, but reduced in size and defective in associative olfactory learning and memory [28]-[31]. Previous work showed that the size and learning defects reflect roles for NF1 in larval and adult neurons respectively, and that loss of *dNf1* is associated with neuronal ERK overactivation [31]. However, no upstream activator of dNf1 regulated Ras-ERK signaling has yet been identified [32]. Here, we present evidence that dAlk functions as an activator of dNf1-controlled neuronal Ras/ERK pathways, responsible for defects in body size determination during larval development and in olfactory learning in adult flies. Our results argue that Alk may provide a therapeutic target for human NF1.

## Results

### dAlk regulates ERK activation in the adult brain

**Figure 1:**
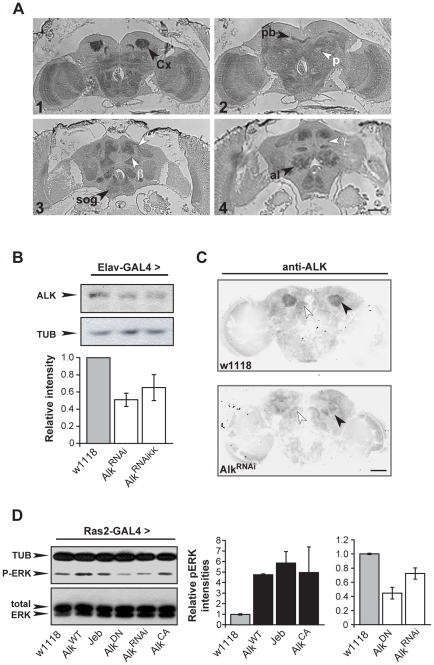
dAlk protein is an active RTK expressed in the adult CNS. (A) Frontal paraffin sections stained with an anti-dAlk antibody, demonstrate accumulation of the receptor in neuropil areas, especially in the Calyx (Cx) (1) the protocerebral bridge (pb) (2) the suboesophageal ganglion (sog) (3,4) and antennal lobe (al) (4) marked with black arrowheads. dAlk is absent from the α, β, and γ lobes of the mushroom bodies and pedunculus (p; white arrowheads) (3,4). Bar = 50 µm. (B) A representative semi-quantitative immunoblot and its quantification illustrate significant reduction of endogenous dAlk upon RNAi-mediated abrogation using two different UAS-*Alk^RNAi^* transgenes (UAS-*Alk^RNAi^* and UAS-*Alk^RNAiKK^*) when driven with *Elav*-Gal4 . ANOVA indicated significant effects of genotype (F_(2,15)_ = 6.51, p<0.01, n = 5). Student's t-test revealed significant differences between controls and the two different UAS-*Alk^RNAi^* lines (p<0.004 and p<0.02 for UAS-*Alk^RNAi^* and UAS-*Alk^RNAiKK^*, respectively). Tubulin levels served as loading control. (C) Immunohistochemical demonstration of anti-dAlk antibody specificity in adult brain. Representative 40 µm confocal z-stacks from brains of adult *w^1118^* (upper panel) and flies expressing the *UAS-Alk^RNAi^* transgene under control of the MB-specific *c772-*Gal4 driver stained with the anti-dAlk antibody. dAlk accumulation within the dendrites (black arrowhead) of wild type flies was highly reduced in the dendrites of flies expressing the *UAS-Alk^RNAi^* transgene. White arrowheads point to the protocerebral bridge where the Gal4 driver is not expressed, showing equal levels of staining between control and experimental flies. Confocal images were acquired within the same range and using the same acquisition settings. They were then converted to grayscale and inverted. Scale bar = 50 µm. (D) Manipulation of dAlk activity in Ras2-expressing neurons affects ERK phosphorylation levels. Representative semi-quantitative immunoblot (left) and its quantification (right) showing alteration of ERK phosphorylation levels upon expression of *dAlk* transgenes in the Ras2-expressing cells. Levels of pERK were significantly higher upon over-expression of *UAS-Alk^WT^* , *UAS-Jeb* and *UAS-Alk^CA^* relative to *w^1118^* control (p = 0.04, p = 0.0053, p = 0.03 respectively, n = 3, Student's t-test). Accordingly, pERK levels were significantly lower upon expression of *UAS-Alk^DN^* or *UAS-Alk^RNAi^* transgenes relative to *w^1118^* control (p = 0.0002 and p = 0.01, n = 3, Student's t-test). The amount of total-ERK protein is not affected. Error bars denote S.E.M.

An expression profiling screen revealed robust expression of *dAlk* mRNA in the adult CNS (data not shown; see Materials and Methods for details). To confirm and extend this finding, adult brain sections were immunostained with an anti-dAlk antibody [33]. These experiments identified prominent dAlk staining throughout the neuropil, whereas cortical areas, including the regions occupied by mushroom body cell bodies, stained to a much lesser extent (Figure 1A1-1A4). Particularly strong staining was observed in mushroom body calyces (Figure 1A1), compared to near background level staining in other mushroom body parts, including the α, β and γ lobes (Figure 1A3 and 1A4), or the pedunculus (Figure 1A2). Other dAlk staining positive CNS structures included the optic lobes (Figure 1A1 and 1A2), the protocerebral bridge (Figure 1A2), the antennal lobes, the suboesophageal ganglion (Figure 1A3 and 1A4), the medial bundle and lateral horns (not shown). In contrast, staining similarly treated sections incubated in the absence of the primary antibody did not reveal any signal, demonstrating the specificity of the anti-dAlk antibody and of the staining pattern (Figure S4A). The intense staining of mushroom body calyces may reflect a specific accumulation of dAlk in dendrites, and the same dendritic accumulation may be reflected by the neuropil staining.

Because homozygous *dAlk* mutants die as early larvae [33], we used two independent RNA-interference (RNAi) transgenes and the TARGET system [34], to abrogate dAlk. Both *Alk^RNAi^* transgenes were specifically expressed in the adult CNS with the *Elav*-Gal4 driver (See Materials and Methods) and resulted in significant reduction of dAlk (Figure 1B). To confirm the specificity of dAlk immunostaining, we next abrogated dAlk expression specifically in mushroom bodies, using the *c772*-Gal4 driver. This resulted in a specific reduction of the dAlk signal in mushroom body calyces, whereas staining in the remaining neuropil remained largely unaffected (Figure 1C). In conjunction with the results in Figure S4A, we conclude that the antibody is highly selective for dAlk and that the *Alk^RNAi^* transgene allows effective ablation of dAlk expression.

Previous work indicated that dAlk can activate ERK in larval imaginal disks [33]. To determine whether the same is true in the adult CNS, we analyzed phospho-ERK (p-ERK) levels in flies expressing various *dAlk* or *Jeb* transgenes. Transgenes designed to enhance dAlk signaling included those encoding wild type (Alk^WT^) [33], a constitutively active truncated form of the protein (Alk^CA^), or the dAlk-activating ligand Jeb [23]. Conversely, the *Alk^RNAi^* transgene, or a transgene encoding a dominant negative *Alk^DN^* mutant [23], were used to block dAlk signaling. In addition to *Elav*, we used the *Ras2*-Gal4 driver [31], which although not as widespread, is also expressed in the majority of CNS neurons (Figure S1C and S1D). Phospho-ERK levels were significantly elevated in head lysates of flies expressing *Alk^WT^*, *Alk^CA^*, or Jeb under Ras2-Gal4 and reduced in flies expressing the interfering *Alk^DN^* or *Alk^RNAi^* transgenes. Thus, dAlk modulates ERK activation in the adult CNS, likely by engaging the Ras/ERK cascade, as previously demonstrated for the vertebrate protein [11].

### dAlk inhibits associative learning in the adult CNS

**Figure 2:**
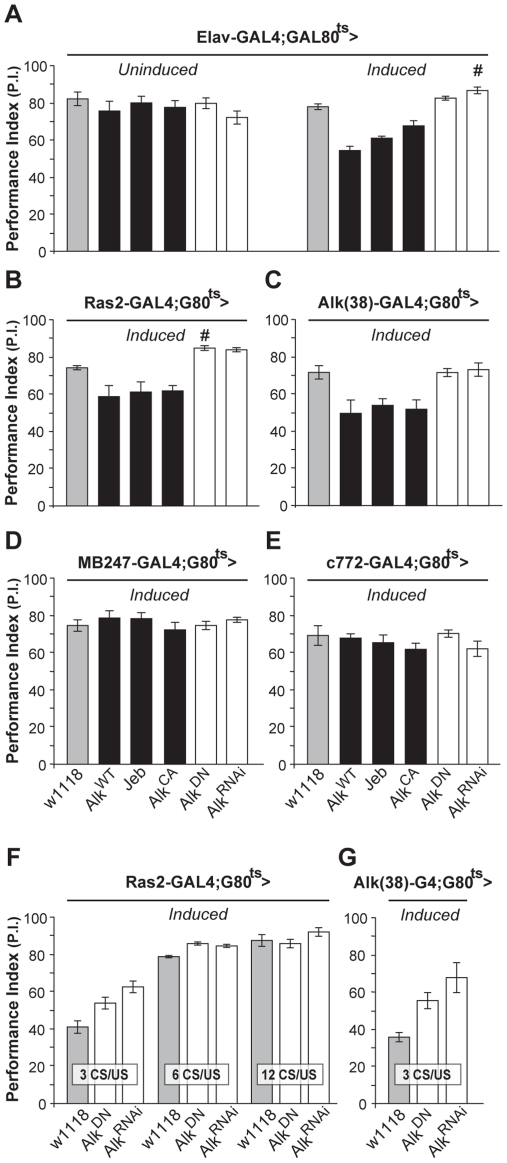
dAlk activity in adult neurons negatively regulates olfactory learning. Mean Performance Indexes ± SEMs are shown after olfactory training with 6 CS/US pairings, unless indicated otherwise. (A) Flies carrying, but not expressing UAS*-dAlk* transgenes (‘*Uninduced*’) performed equally well with controls (F_(5,40)_ = 0.94, p>0.46, n≥6 for all genotypes). Pan-neuronal transgene expression (‘*Induced*’) affected learning, as indicated by ANOVA (F_(5,41)_ = 23.72, p<0.0001, n≥6). Pairwise comparisons demonstrated highly significant differences in the performance of animals expressing *Alk^WT^*, *Jeb* and *Alk^CA^* compared to heterozygous driver carrying control flies (*w^1118^*). (B and C) dAlk manipulations also affected learning in *Ras2*-Gal4 and *Alk(38)*-Gal4 expressing neurons (ANOVA F_(5,45)_ = 16.8, p<0.0001, n≥6 and F_(5,36)_ = 7.142, p<0.0002, n = 6 respectively). Highly significant differences in the performance of *Alk^WT^*, *Jeb* and *Alk^CA^-*expressing animals were documented in comparison to control flies. (D and E) Expression of dAlk transgenes within the MB using *MB247* and *c772* drivers did not affect learning (F_(5,42)_  = 0.70, p>0.62, n≥6 for *MB247* and F_(5,45)_ = 1, p>0.42, n≥7 for *c772*). Significantly enhanced learning relative to controls upon abrogation of dAlk activity is denoted by (#). (F) Characterization of the learning enhancement phenotype upon down-regulation of dAlk signaling in Ras2-expressing cells. Significant effects of genotype were found after less intense training conditions (3 and 6 CS/US pairings) but not after 12 CS/US training, indicating a plateau effect (ANOVA F_(2,20)_ = 12.15, p<0.0005, F_(2,22)_ = 27.35, p<0.0001, F_(2,20)_  = 1.38, p>0.27 for 3 CS/US, 6 CS/US and 12 CS/US respectively, n = 6–8 for all genotypes). Planned pairwise comparisons indicated significant differences between controls and flies with down-regulated dAlk when trained with 3 CS/US pairings (p<0.0001 and p<0.013 for UAS*-Alk^RNAi^* and UAS*-Alk^DN^*, respectively) and 6 CS/US pairings (p<0.0001 for UAS*-Alk^RNAi^* and UAS*-Alk^DN^*). (G) Down-regulation of dAlk in otherwise wild type cells enhanced learning performance under low training conditions with 3 CS/US pairings. ANOVA indicated significant effects of genotype (F_(2,16)_ = 8.09, p<0.0052, n>5). Planned pairwise comparisons indicated significant differences between controls and flies with down-regulated dAlk signaling in Alk-expressing cells (p<0.0016 and p<0.02 for UAS*-Alk^RNAi^* and UAS*-Alk^DN^*, respectively).

To investigate whether dAlk plays a role in associative learning as suggested by its presence in the mushroom bodies, we again used the TARGET system to modulate dAlk expression specifically in the adult CNS, thus avoiding potential defects stemming from its known developmental roles [22]–[24]. Surprisingly, adult-specific pan-neuronal expression of wild type dAlk, its constitutively active Alk^CA^ mutant, or its activating ligand Jeb, caused significant deficits in associative olfactory learning (Figure 2A-Induced). Because the only known function of Jeb is to activate dAlk, it appears unlikely that the observed learning impairment is a non-specific consequence of expressing the transgene ectopically. Moreover, it is unlikely that the attenuated learning upon dAlk over-expression or over-activation is due to ectopic expression, because the same effect was observed in response to transgenic Jeb elevation. Interestingly, interfering with endogenous dAlk activity with the dominant negative protein, or attenuating its levels in the CNS by RNAi, did not precipitate deficits, but rather reproducibly elevated performance (Figure 2A, open bars, # p = 0.0016). Learning deficits were not observed in flies raised at the restrictive temperature (Figure 2A, ‘Uninduced’), and task-relevant olfactory responses and shock reactivity were normal (Table S1).

Similar learning deficits were observed upon increasing dAlk expression or activity using the *Ras2* neuronal driver (Figure 2B). Again, abrogating endogenous dAlk by expressing *UAS-Alk^RNAi^*, or attenuating its activity with Alk^DN^ in Ras2-positive neurons yielded a modest, but significant (# p = 0.01) learning enhancement (Figure 2B). To ascertain that these effects were not a consequence of ectopic transgene expression, we generated a novel Gal4 driver *Alk(38)*-Gal4. The pattern of *Alk(38)*-Gal4 expression did not overlap precisely with endogenous dAlk distribution in few tissues and cell types as commonly observed with reporter expression and may in part reflect differences in the localization of dAlk and the mCD8-GFP reporter in the membranes. However, *Alk(38)*-Gal4 was abundantly expressed and largely recapitulated the endogenous dAlk distribution in larval and adult CNS (Figure S1A and S1B). *Alk(38)*-Gal4-mediated adult-specific induction of the above mentioned transgenes yielded similar results to those obtained with *Elav* and *Ras2* (Figure 2C). Therefore, increased dAlk levels or signaling impairs olfactory learning, whereas its attenuation improves it.

Both *Alk(38)* and *Ras2*-Gal4 are expressed in the MBs, neurons that contain an abundance of dAlk in their dendrites (calyces). Thus, we were surprised to find that expressing *dAlk* or *Jeb* transgenes with the *MB247* and *c772*-Gal4 MB drivers did not affect associative learning (Figure 2D and 2E). It is unlikely that the lack of effect is due to limited expression in the approximately 2000 MB neurons located in each adult brain hemisphere. In fact, the *MB247* and *c772* drivers are expressed in 1600 and 1800 MB neurons respectively and *c772* in particular is also expressed in α3′/β3′ cells [35]. Therefore, despite its obvious presence in the calyces, dAlk does not appear to play a role in olfactory associative learning in most, if not all, MB neurons.

Because enhanced learning is an uncommon phenotype [36], we sought additional substantiation that dAlk inhibition caused this effect. To increase assay resolution, we limited the number of conditioned/unconditioned stimulus (CS/US) pairings, as described previously [9]. Limited training with just 3 pairings also resulted in enhanced learning when Alk was abrogated with the *Ras2* (Figure 2F), or *Alk(38)*-Gal4 drivers (Figure 2G), validating this finding. Enhanced learning was also apparent after training with 6, but not after the more intense 12 pairing training, suggestive of a performance “plateau” [9]. We conclude that inhibiting dAlk enhances performance after suboptimal training, apparently by increasing learning per CS/US pairing. This increased learning rate allows dAlk inhibited flies to reach a performance plateau faster than controls. Thus, dAlk appears to be a limiting negative regulator of olfactory associative learning and surprisingly this function seems to involve neurons outside the mushroom bodies.

### dAlk activity in the developing CNS regulates body size

**Figure 3:**
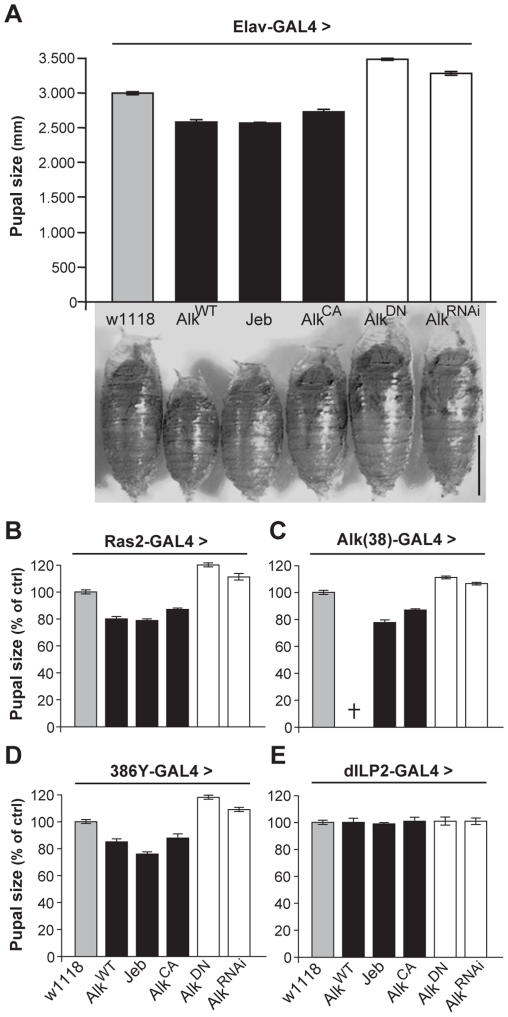
dAlk activity in Ras2-expressing neurons controls organism size. (A) Pan-neuronal activation of dAlk signaling by *Elav*-Gal4-driven expression of *Alk^WT^*, *Jeb* and *Alk^CA^* transgenes reduces pupal size, whereas abrogation of dAlk function with *Alk^DN^* or *Alk^RNAi^* expression increases pupal size relative to heterozygous driver controls (*w^1118^*) (n = 20 for each genotype). Representative pupae of each genotype are shown (Bar = 1 mm). Planned pair-wise comparisons between control and transgenic flies revealed significant differences for all genotypes (ANOVA F_(5,120)_ = 217.62, p<0.0001). (B-E) dAlk controls size in Ras2-expressing, dAlk-expressing and neuropeptidergic cells, but not in insulin-producing cells. Significant effects of genotype on size were found with *Ras2*-Gal4 (n = 50), *Alk(38)*-Gal4 (n = 30–31) and *386Y*-Gal4 (n = 16–17) (ANOVA F_(5,300)_ = 460.97, F_(5,99)_ = 230.03, F_(5,154)_ = 406.05 respectively, p<0.0001), but not with *dILP2*-Gal4 (F_(5,131)_ = 0.82, p = 0.53). Cross denotes larval lethality. Error bars denote S.E.M.

In contrast to the normal-sized animals obtained with use of the TARGET system, we noted that pan-neuronal expression of wild-type dAlk, or of the constitutively active *dAlk^CA^* throughout development yielded ∼10%–15% smaller pupae compared to isogenic controls (Figure 3A). Similar results were obtained by over-expression of Jeb, which again argues against an artifact caused by ectopic dAlk expression, since Jeb should only activate its endogenous dAlk receptor. Further arguing against a non-specific effect, abrogation of endogenous dAlk activity by pan-neuronal expression of the dominant-negative *dAlk^DN^* or *Alk^RNAi^* transgenes yielded pupae that were ∼10%–15% larger than wild-type (Figure 3A). Except for these conspicuous size differences, normally patterned and viable adults emerged from these pupae (Figure S2). Driving the *dAlk* and *Jeb* transgenes with *Ras2* and *Alk(38)*-Gal4 caused similar size changes (Figure 3B and 3C). Notably, increasing the levels of wild type dAlk in neurons normally expressing the protein with the *Alk(38)* driver resulted in larval lethality, suggesting that animals below a certain size threshold are not viable, possibly because of neuromusculature defects [22], [24].

To further define the role of dAlk in body size determination, we sought to identify the types of cells in the larval CNS where it is required for this novel function. Therefore, we used 29 additional Gal4-drivers to drive the *dAlk* transgenes in various CNS cell types throughout development (Table S2). Body size was altered by manipulating dAlk expression or activity in cells marked by the peptidergic drivers *386Y*-Gal4 (Figure 3D), *c929-*Gal4 and the cholinergic *Cha-*Gal4 (Table S2). In contrast, increasing dAlk activity in glia, MBs, dopaminergic and GABAergic neurons did not affect pupal size (Table S2). Interestingly, transgenic Jeb expression in glial cells, GABAergic and dopaminergic neurons and the potentially peptidergic *c316*-Gal4-marked DPM neurons [37], resulted in small pupae. These results are consistent with a paracrine mode of action for Jeb as previously suggested [22], [38]. Significantly, driving dAlk in Insulin-producing cells (IPCs), known to be involved in Drosophila body size determination [39], [40], also had no effect (Figure 3E). This suggests that dAlk operates in a different, potentially independent size determination system than that requiring Insulin.

**Figure 4:**
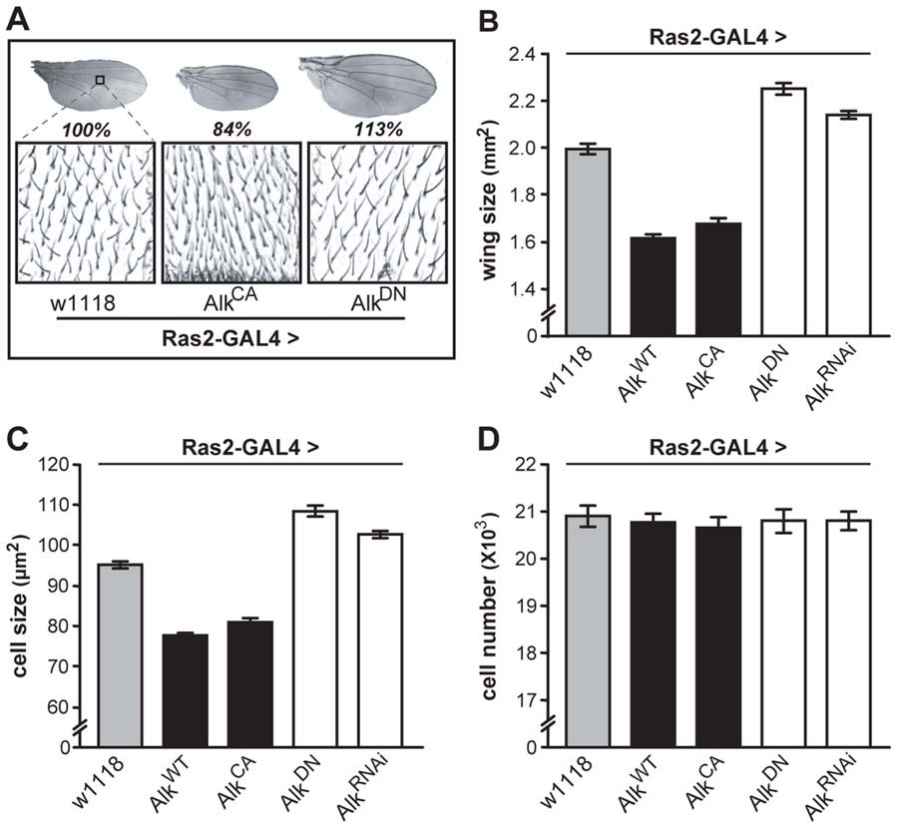
dAlk activity in Ras2-expressing cells affects organism size through changes in cell size and not cell number. (A) Representative pictures of wings isolated from female flies of the indicated genotype. The magnified areas show wing hairs, arising from single wing-blade intervein cells located between veins 3 and 4 on the dorsal wing blade, up to the posterior cross vein. (B) Measurements of wing surface areas shows significant differences in total wing size for each genotype (ANOVA F_(4,65)_  = 197.74, p<0.0001, n≥12). Calculated cell size (C) and total number (D) of intervein cells reveal changes in adult wing size correspond to significant changes in cell size (ANOVA F_(4,65)_  = 186.32, p<0.0001, n≥12) and not cell number (F_(4,65)_  = 0.14, p>0.96, n≥12). Error bars denote S.E.M.

Measuring wing surface areas confirmed that flies with altered body size possess proportionally sized wings (Figure 4A and 4B). This likely reflects an altered size of the larval wing disk, and agrees with the notion that dAlk is required in the larval CNS to determine body size in a non-cell autonomous manner. To determine whether size differences reflect changes in cell size and/or cell number, we took advantage of the fact that each wing cell produces a single hair, which provides a convenient way of determining wing cell densities [41]. Comparing the cell densities of wings from *Ras2*-Gal4 driven transgenic flies to those of wild type controls, we found that size alterations are largely due to changes in cell size (Figure 4C) and not cell number (Figure 4D). Collectively, these data demonstrate that dAlk and Jeb are novel non-cell-autonomous regulators of organismal growth, affecting cell size but not proliferation.

**Figure 5:**
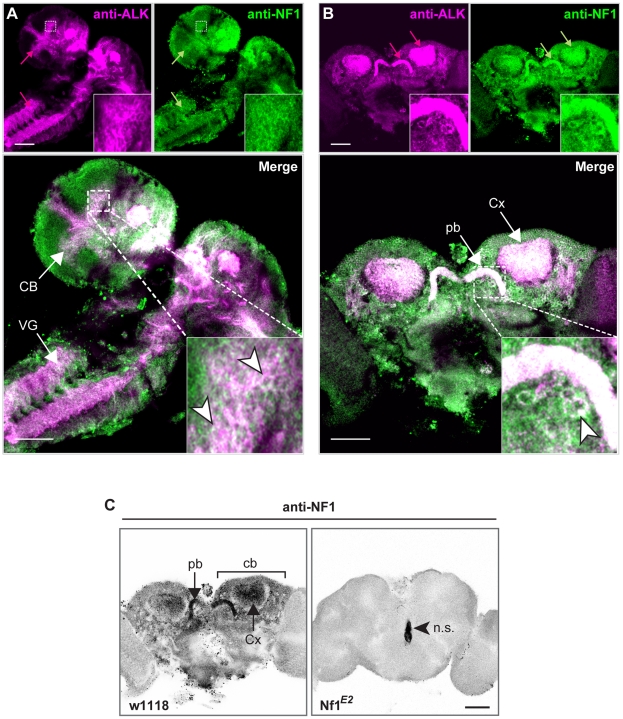
dAlk and dNF1 extensively colocalize in larval and adult CNS. dAlk and dNF1 co-localize in larval CNS (A) or adult brain (B). 10 µm confocal z-stacks of third instar larval CNS (A) or adult brain (B) were acquired at the level of the calyces and visualized with anti-dAlk (purple) and anti-dNf1 (green). Colocalization of dAlk and dNf1 immunofluorescence (Alk+Nf1) is shown in white. Inset: higher magnification of the hatched boxes, showing colocalization in single cells (arrowheads). Bar = 50 µm. (C) Immunohistochemical demonstration of anti-dNf1 antibody specificity. Representative optical sections at the level of the calyces from brains of adult *w^1118^* (left column) and *Nf1^E2^-null* mutant flies (right column) stained with the anti-dNf1 monoclonal antibody. dNf1 protein clearly accumulates within the dendrites (calyces, Cx) and cell bodies (cb) of mushroom body neurons, as well as in the protocerebral bridge (pb). It is also widely expressed in the neuropil, above general background staining. In contrast, brains of *Nf1^E2^-null* mutants are clearly devoid of staining, revealing the specificity of the anti-Nf1 antibody. Arrowhead points to area stained non-specifically (n.s.). Confocal images were acquired at the same section levels and using the same settings. They were then converted to grayscale and inverted. Scale bars = 50 µm.

Growth of insects occurs exclusively during larval development. Therefore, it was not surprising that pan-neuronal expression of *dAlk* specifically in adult flies with TARGET did not modify their size. Rather, it appears that the size-related function involves larval neuroendocrine and cholinergic neurons. This conclusion is supported by the spatial specificity of Gal4 drivers that modify size (Table S2), as well as by the prominent dAlk immunostaining (Figure 5A) in discrete parts of the larval ventral ganglion and the central brain where neuroendocrine and cholinergic neurons reside [42]. Notably, the *Alk(38)* driver is also expressed in these areas of the larval ventral ganglion (Figure S1A).

### Developmental attenuation of dAlk signaling in the CNS reverses the body size deficits of *dNf1* mutants

The size and learning defects observed upon dAlk over-activation are highly reminiscent of phenotypes exhibited by Drosophila *dNf1* mutants [26], [29]–[31], [43]. An important goal of Neurofibromatosis 1 research has been to identify upstream activators of NF1 regulated Ras signaling, which might be exploited as potential therapeutic targets. To test whether dAlk plays such a role, we compared dAlk and dNf1 expression patterns, and we investigated whether the two genes interact genetically.

**Figure 6:**
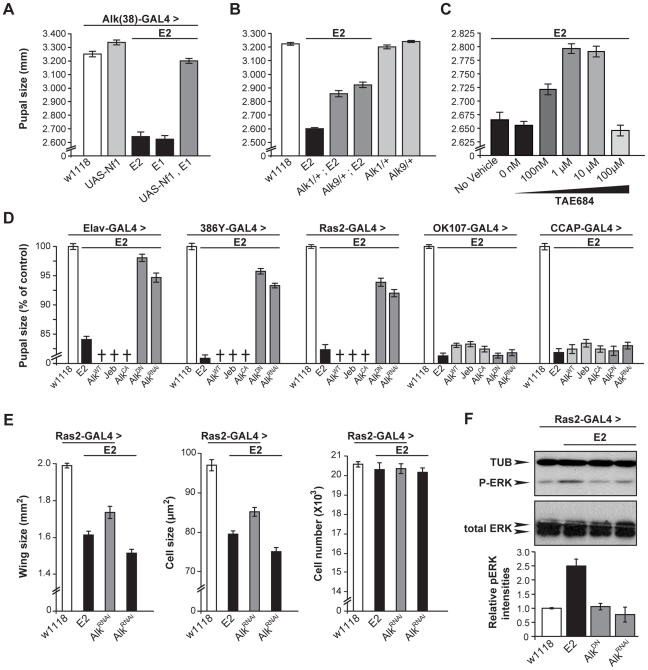
dAlk interacts with dNf1 to control pupal size and ERK activation. (A) *Alk(38)-*Gal4 over-expression of UAS*-Nf1* in *Nf1^E1/E2^* null mutants rescues pupal size (ANOVA F_(4,102)_  = 240.88, p<0.0001, n≥19). Heteroallelic *Nf1^E1/E2^* mutant flies were used for ease of genetic manipulations. Differences between *Alk(38)-*Gal4 over-expression of UAS*-Nf1* in *Nf1^E1/E2^* background and *Alk(38)-*Gal4/+ control (p = 0.10) were not significant. (B) *Alk^1^* and *Alk^9^* heterozygous mutations interact genetically with *Nf1^E2^*. ANOVA indicated significant differences between *Alk^1^*/+;*Nf1^E2^*, or *Alk^9^*/+;*Nf1^E2^* and *Nf1^E2^* pupal size (F_(5,195)_  = 390.63, p<0.0001, n≥25). (C) The Alk inhibitor TAE684 ameliorates *Nf1^E2^* pupal size defects. ANOVA indicated significant effects of inhibitor concentration (F_(4,565)_ = 82.42, p<0.0001, n≥100). (D) dAlk down-regulation targeted pan-neuronally, in neuroendocrine and Ras2-expressing cells rescues *Nf1^E2^* pupal size. Significant differences in pupal length were uncovered between *Nf1^E2^* mutants expressing UAS-*Alk^DN^* or UAS-*Alk^RNAi^* and *Nf1^E2^* alone (F_(3,133)_  = 176.97, F_(3,157)_  = 388.14, F_(3,135)_  = 207.60 for *Elav*-Gal4, *386Y*- Gal4, and *Ras2*-Gal4 respectively, n≥30 , p<0.0001). Rescue was not observed with MB (*OK107*-Gal4), or neuropeptidergic (*CCAP*-Gal4) drivers. Crosses denote lethality. (E) Cell size, but not cell number increases upon rescue of the size defects of *Nf1^E2^* homozygotes by dAlk inhibition. Amelioration of *Nf1^E2^* homozygous mutant size deficits by RNAi-mediated abrogation of endogenous dAlk targeted in Ras2-expressing cells is attributed specifically to increase of cell size and not of cell proliferation. ANOVA indicated significant effects of genotype on wing size (F_(3,55)_ = 99.72, p<0.0001, n≥13) and cell size (F_(3,55)_ = 120.88, p<0.0001, n≥13). No significant effects were uncovered on total cell number (F_(3,55)_ = 0.51, p<0.67, n≥13). Planned pairwise comparisons between *Nf1^E2^* flies without compromised dAlk expression and *Nf1^E2^* flies with abrogated dAlk expression in Ras2-expressing cells showed significant differences on wing size and cell size indicating rescue (p<0.0001 for all comparisons). (F) Attenuation of dAlk signaling restores ERK overactivation in *Nf1^E2^* mutants. Representative immunoblot and semi-quantification (histograms below) of four such blots. The levels of phosphorylated ERK in *Nf1^E2^* mutants were significantly different from *w^1118^* control (p<0.0001, n = 4, Student's t-test) indicating elevation of pERK levels. In contrast, *Nf1^E2^* larvae with compromised dAlk activity have normal pERK levels (p = 0.84 for *UAS-Alk^DN^* and p = 0.42 for *UAS-Alk^RNAi^* respectively) demonstrating restoration of ERK phosphorylation of *Nf1^E2^*-null mutants to normal upon abrogation of dAlk levels or function. Error bars denote S.E.M.

In order for dAlk to function as an upstream activator of dNf1 regulated Ras signals, both proteins should at least partially colocalize. Using an antibody specific to dNf1 (Figure 5C), we found that both proteins are broadly distributed in the larval CNS, and colocalize extensively in the larval ventral nerve cord, the brain lobes and particularly in the larval mushroom body calyces and the developing adult visual system (Figure 5A). Both proteins are also broadly present throughout the adult brain and show similar extensive colocalization in mushroom body calyces, the protocerebral bridge, mushroom body satellite neuropil and ventral bodies (Figure 5B). Furthermore, both proteins appeared in close proximity with the cell membrane in different neuronal types (Figure 5 insets). To functionally test the colocalization of the two proteins, we took advantage of the fact that loss of dNf1 also results in a non-cell autonomous decrease in cell size yielding overall smaller pupae [30], [31] and adults (Figure S2). Targeted re-expression of *dNf1* in *Alk(38)*-Gal4-expressing cells reversed the size deficit of *Nf1-*null mutants (Figure 6A) indicating that dNf1 is indeed required in dAlk-expressing cells. Thus, dAlk and dNf1 show widespread and extensively overlapping expression in both the larval and adult CNS. Moreover, both proteins appear to function in overlapping neuronal populations to non-autonomously regulate organismal size.

To substantiate this conclusion, we evaluated functional links between dAlk and dNf1 using genetic epistasis. Pupal size in *Alk^1^-null* and *Alk^9^-kinase-dead* heterozygotes is normal (Figure 6B). However, heterozygosity for either *Alk* mutant allele increased significantly the small size of *Nf1^E2^* homozygotes (Figure 6B). This phenotypic suppression was a consequence of increased cell size and not cell number (Figure S3A). Importantly, additional support for the hypothesis that dAlk and dNf1 function in the same growth regulating pathway was provided by pharmacological abrogation of dAlk with the selective inhibitor TAE684 [44]. Feeding 1–10 µM TAE684 to homozygous null *Nf1^E2^* larvae throughout development rescued their size deficit. No phenotypic rescue was observed at 100 µΜ, indicating a sharp concentration optimum and perhaps reflecting an off-target effect at the higher concentration (Figure 6C). Therefore, dAlk and dNf1 interact genetically to determine pupal size.

To define more precisely the cells where dAlk and dNf1 function for size determination, *Alk^WT^, Alk^CA^* and *Jeb* transgenes were expressed with *Elav*, *Ras2* and the more restricted *386Y*-Gal4 in *Nf1^E2^* mutants. All these manipulations caused larval lethality, suggesting that further reduction of the already small size of *Nf1^E2^* is not tolerated (crosses in Figure 6D). In agreement with this interpretation, pan-neuronal expression of *Alk^WT^* and *Alk^CA^* in *Nf1^E2/+^* heterozygotes was also lethal, whereas *Jeb* expression enhanced their slight size reduction to 70% of controls. The *Nf1^E2/+^* small size was also further reduced upon *Jeb* expression with *Ras2* and *386Y*, but not with the MB-driver *OK107*-Gal4 (Figure S3B). Conversely, abrogation of dAlk signaling with *Alk^DN^* or *Alk^RNAi^* resulted in *Nf1^E2^* homozygotes with significantly larger size, which approached control levels if driven with *Elav*. Driving the same transgenes with *Ras2* and *386Y*-Gal4 also increased pupal size, but to a lesser extent (Figure 6D). Similar attenuation of dAlk activity in *Nf1^E2/+^* heterozygotes rescued their smaller size completely (Figure S3B). Importantly, rescue of the *Nf1^E2^* small size by dAlk abrogation is the result of increased cell size and not cell number (Figure 6E). These results provide additional support for the proposed functional interaction of the two proteins in size determination. In contrast, dAlk abrogation in MB neuroblasts and neurons with *OK107*-Gal4, or in CCAP-producing neurons with *CCAP*-Gal4, did not alter *Nf1^E2^* size (Figure 6D).

Increased ERK activity as a result of dNf1 loss in larval Ras2-expressing cells has been proposed to result in the reduced body size in mutant homozygotes [31]. In agreement with this hypothesis targeted expression of an activated form of ERK (UAS-*rl^sem^*) [45] with *Ras2*-Gal4, yielded smaller animals with wings that consisted of smaller, but not fewer cells (Figure S3C). If our hypothesis that dAlk activates dNf1-regulated Ras/ERK signaling is correct, then attenuating dAlk activity should reduce the elevated neuronal phospho-ERK levels in *Nf1^E2^* homozygotes. Indeed, abrogation of dAlk levels or activity in *Ras2*-expressing neurons of *Nf1^E2^* homozygotes, restored phospho-ERK to control levels (Figure 6F). Collectively, these results indicate that neuronal ERK activity levels control Drosophila size, and that attenuation of ERK over-activation by genetic or pharmacological reduction of dAlk activity rescues the small size phenotype of *dNf1* mutants.

### Attenuation of dAlk rescues the deficient learning of *dNf1* mutants

Since dAlk (Figure 2) and dNf1 [29] both affect associative learning independent of their developmental roles, we investigated whether the two proteins interact in neurons mediating this process. Immunostaining revealed that dNf1 is distributed in the adult brain in a pattern highly overlapping that of dAlk, exhibiting coincident staining in mushroom body calyces and the protocerebral bridge, among other regions (Figure 5B). Interestingly, much like dAlk, dNf1 also appears conspicuously absent from the pedunculus and lobes of the MBs (Figure S4). Others recently reported the presence of *dNf1* transcripts in mushroom bodies by *in situ* hybridization [28]. Our results confirm and extend this work, by providing the first documentation of the spatial distribution of dNf1 protein in the adult brain including the MBs.

**Figure 7:**
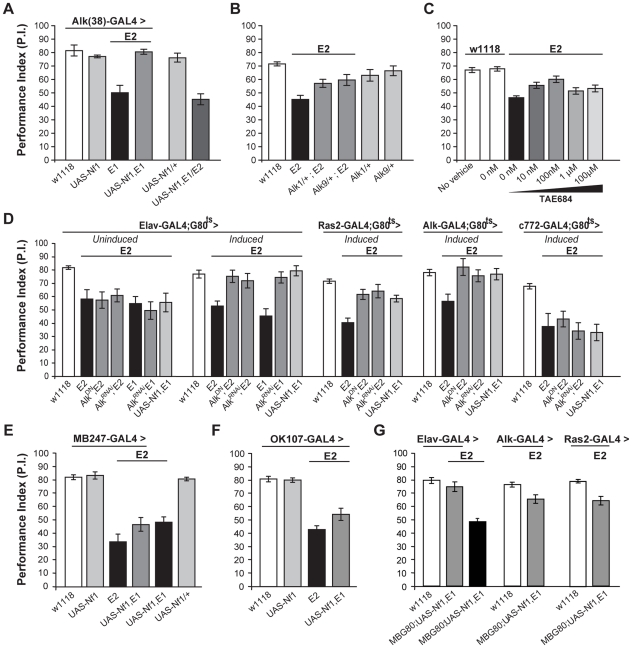
dAlk interacts with dNf1 to regulate learning. (A) *Alk(38*)-Gal4 expression of UAS*-Nf1* in *Nf1^E1/E2^* null mutants rescues learning. Heteroallelic *Nf1^E1/E2^* mutant flies were used for ease of genetic manipulations. ANOVA indicated significant effects of genotype (F_(5,40)_ = 20.36, p<0.0001, n≥6), whereas no significant differences between *Alk(38)*-Gal4 driven UAS*-Nf1* in *Nf1^E1/E2^* mutants and *Alk(38)*-Gal4/+ controls were found. (B) *Alk^1^* or *Alk^9^* heterozygous mutant alleles and *Nf1^E2^* interact genetically to rescue learning. The performances of *Alk^1^*/+;*Nf1^E2^*, *Alk^9^*/+;*Nf1^E2^* and *Nf1^E2^* were significantly different (ANOVA: F_(5,79)_  = 8.78, p<0.0001, n≥12). (C) The Alk inhibitor TAE684 rescues *Nf1^E2^* learning defects (ANOVA: F_(6,81)_ = 18,60, p<0.0001, n≥10). Planned comparisons (shown in Table S3) showed significant differences between 0 nM and 10 nM or 100 nM concentrations. (D) Adult induced dAlk down-regulation pan-neuronally or in Ras2- and Alk(38)- expressing cells rescues *Nf1^E2^* learning defects. ANOVA indicated significant effects of *Nf1^E2^* mutants expressing UAS-*Alk^DN^* or UAS-*Alk^RNAi^* when compared to *Nf1^E2^* alone (F_(6,55)_  = 9.49, p<0.0001, n≥7 for *Elav*-Gal4;Gal80^ts^ (‘*Induced*’), F_(4,41)_ = 15.89, p<0.0001, n≥8 for *Ras2*-Gal4;Gal80^ts^, F_(4,38)_ = 4.15, p<0.0078, n≥6 for *Alk(38)*-Gal4;Gal80^ts^ respectively). No rescue is observed upon bearing but not expressing the transgenes pan-neuronally (‘*Uninduced*’), or using the MB-specific *c772*-Gal4;G80^ts^ driver. (E) Over-expression of a wild-type dNf1 transgene (UAS*-Nf1*) in the MBs using *MB247*-Gal4 fails to restore learning in *Nf1* mutants. ANOVA indicated significant effect of genotype (F_(5,46)_  = 23.31, p<0.0001, n≥7 for all genotypes). Planned pairwise comparisons indicated significant differences between *Nf1^E2^* mutant flies harboring the *MB247*-Gal4 driver, UAS*-Nf1* transgene, or mutant flies overexpressing the wild-type dNf1 transgene in the MBs and heterozygous driver controls (p<0.0001 for all comparisons). (F) Over-expression of a wild-type dNf1 transgene (UAS*-Nf1*) in mushroom body neurons using *OK107*-Gal4 during development fails to restore performance of *Nf1* mutants. ANOVA indicated significant effect of genotype (F_(3,33)_  = 37.72, p<0.0001, n≥8 for all genotypes). Planned pairwise comparisons indicated significant differences between *Nf1^E2^* mutant flies harboring the *OK107*-Gal4 driver, or mutant flies overexpressing the wild-type dNf1 transgene in *OK107-*marked mushroom body neurons and heterozygous driver controls (p<0.0001 for all comparisons). (G) Pan-neuronal expression of a wild-type dNf1 transgene (UAS*-Nf1*) or in Alk- and Ras2- expressing neurons with the exclusion of the MBS restores learning in *Nf1^E1/E2^* mutant flies. ANOVA indicated significant effect of genotype (F _(6,66)_  = 20.13, p<0.0001, n≥8 for all genotypes). Planned pairwise comparisons indicated significant differences between *Nf1^E1/E2^* flies harboring the MB-Gal80 (MBG80) and UAS-*Nf1* transgenes and mutant flies overexpressing the transgene pan-neuronally, as well as in Alk- positive and Ras2-positive cells excluding mushroom body neurons (p<0.0001). The performance of Elav;MBG80;UAS*-Nf1*,*Nf1^E1/E2^* flies was not statistically different from their respective heterozygous driver control indicating full rescue (p = 0.26), contrary to the performance of flies overexpressing the transgene in Alk- positive and Ras2- positive cells, indicating partial rescue (p = 0.003 and p = 0.0003 for *Alk(38)*-Gal4 and *Ras2*-Gal4 respectively). Error bars denote S.E.M.

As previously reported for the *dNf1^P1^* and *dNf1^P2^* alleles [29], *Nf1^E1^* and *Nf1^E2^* mutant homozygotes and *Nf1^E1/E2^* heteroallelics also exhibit significant associative learning defects (Figure S5A). In fact, upon training with 3 CS/US pairings, even the heterozygous null mutants exhibited a learning defect (Figure S5B). In support of the notion that dNf1 and dAlk function in the same cells to regulate learning, the learning defect of *dNf1^E1/E2^* animals was fully rescued by targeted re-expression of dNf1 within *Alk(38)*-Gal4-expressing cells (Figure 7A). To investigate whether dAlk-mediated signals are potentially regulated by dNf1 presumably within *Alk(38)*-Gal4-expressing cells in the adult CNS, we attempted to dominantly suppress the learning deficit of *Nf1^E2^* homozygotes by reducing the dAlk gene dosage, using the *Alk^1^* null and the *Alk^9^* kinase-dead alleles. Either mutant allele improved the associative learning of *Nf1^E2^* mutants (Figure 7B), consistent with the hypothesis that dAlk and dNf1 interact to mediate the process. Importantly, acute inhibition of dAlk with TAE684 [44] in adult *Nf1^E2^* homozygotes nearly eliminated their learning deficit (Figure 7G, 10 nM and 100 nM, p = 0.0013 and p<0.0001 respectively, compared to vehicle-fed *Nf1^E2^* flies). Similar to what was observed in size rescue experiments, the pharmacological response to acute TAE684 administration had a clear optimum in the 10-100 nM range, whereas higher doses were ineffective (Figure 7C). This pharmacological evidence strongly suggests that dAlk-mediated signals engage dNf1 and are required for normal associative learning in flies.

To determine the neurons where these proteins are required for associative learning, dAlk was attenuated with *Alk^DN^* and *Alk^RNAi^* in defined neuronal groups specifically in adult *Nf1^E2^* homozygotes (Figure 7D). If these transgenes remained uninduced, learning was defective in these small-sized *Nf1^E1/E2^* adults (Figure 7D). However, pan-neuronal expression under *Elav*-Gal4 completely reversed the learning deficit of *Nf1^E1/E2^*, and this was also attained with the *Alk(38)* and to a lesser extent with the *Ras2* driver (Figure 7D). Similar results were also obtained with a second independent UAS-*Alk^RNAi^* transgene (Figure S5C).

Consistent with the data indicating a function for dAlk in learning outside the MBs, rescue of the *Nf1* learning deficit was not observed with *c772*-Gal4 (Figure 7D). Importantly, over-expression of a wild-type *dNf1* transgene in mushroom bodies also failed to improve performance of *Nf1* mutants (Figure 7D last graph, last column, p<0.0001 compared to control), arguing that learning requiring dAlk and dNf1 does not directly involve these neurons. This result was confirmed with additional mushroom body drivers, *MB247* (Figure 7E) and *OK107* (Figure 7F). However, as with *Elav* [46], both *Alk(38)* and *Ras2-*Gal4 which rescue the learning deficit are expressed in the MBs (Figure S1). Therefore, to unequivocally determine that dNf1 function is not required within the MBs for associative learning, we performed additional experiments using these drivers in combination with *MB*-Gal80, which constitutively prevents transgene expression specifically in the mushroom bodies [47]. Transgenic expression of the *dNf1* transgene under the pan-neuronal *Elav* driver resulted in complete rescue of the associative learning deficits of *Nf1^E1/E2^* animals (Figure 7G left side). Learning of *Nf1^E1/E2^* animals was also significantly improved when UAS-*dNf1* was driven with *Alk(38)* or *Ras2*-Gal4, consistent with the notion that dAlk and dNf1 interact genetically within neurons expressing these drivers to mediate associative learning (Figure 7G). These results confirm that dNf1 is not required within the MBs for normal associative learning. However, although significantly improved, learning of *Nf1^E1/E2^* flies with UAS-*dNf1* driven with *Alk(38)* and *Ras2*-Gal4 did not attain control levels as under *Elav-Gal4*. This indicates that *Ras2* and *Alk(38)*-Gal4 may not be expressed in a subset of *Elav-Gal4* positive neurons where dNf1 is necessary for normal associative learning and complete rescue of the learning deficits in *Nf1^E1/E2^* animals. Alternatively, the reduced rescue may reflect the lower expression level of *Alk(38)* and *Ras2*-Gal4 compared with that of *Elav*. In addition, full rescue of the *Nf1^E1/E2^* learning deficit was observed upon adult-specific dAlk attenuation in *Alk(38)*-expressing neurons (Figure 7D), but not upon expressing a *dNf1* transgene in the same neurons. A potential explanation of this is that although dAlk functions outside the MBs, dNf1 may be required both outside and inside these neurons for normal associative learning. Experiments to resolve this issue and to define the minimal neuronal subset for full rescue of the deficit are currently ongoing.

## Discussion

The prominent expression of Alk in the mammalian and Drosophila CNS [33], [48], [49] and presence of the dAlk ligand Jeb in the embryonic fly CNS [38], provided the first indication that Alk and Jeb likely participate in the development of the nervous system. Subsequent *in vitro* studies demonstrated that Alk promoted neuronal differentiation of PC12 or neuroblastoma cell lines, and work in *C. elegans* implicated its Alk ortholog, *scd-2*, in the inhibition of presynaptic neuronal differentiation *in vivo* [11]. Several functions have also been attributed to Drosophila dAlk, including roles in the specification of the intestinal musculature [20]–[22], in retinal axon targeting [23], and in signaling at the larval neuromuscular junction [24]. Our results establish two novel *in vivo* functions for the Drosophila dAlk/Jeb receptor/ligand pair, in the regulation of organismal growth and associative learning.

### Roles for dAlk and dNf1 in body size regulation

The results presented here lead us to hypothesize that dAlk and dNf1 have opposite roles in controlling neuronal ERK activity during larval development, and therefore determine overall organismal size in a non-cell autonomous manner. In support of our hypothesis, dAlk and dNf1 co-localize extensively in larval neurons, both proteins control ERK activity, and both modulate growth by regulating cell size. In agreement with this conclusion, transgenic neuronal expression of the constitutively active ERK, Rl^SEM^, is sufficient to reduce Drosophila size.

dAlk is the second active RTK implicated in Drosophila growth control. Previous work demonstrated that the fly homolog of the insulin/IGF1 receptor dInr, regulates both body and organ size [40], [41], [50]. In peripheral tissues, dInr is activated by a family of Insulin-like proteins (dILPs), leading to the activation of the IRS (Chico), PI(3)K (Dp110), PTEN (dPTEN), and Akt/PKB, (dAkt1/dPKB), signaling pathway. Ablating the Insulin Producing Cells (IPCs), or silencing the function of dInr pathway components in the larval CNS resulted in severe growth defects [40], [41], [50]. Notwithstanding the similar growth phenotypes, several lines of evidence argue that dAlk and dInr control growth in fundamentally different ways. Most importantly, organismal growth is impaired when dInr activity or signaling is reduced, whereas a similar phenotype is observed upon dAlk activation. Secondly, dAlk affects organism growth by specifically regulating cell size in a non cell-autonomous manner. In contrast, dInr signaling affects both cell size and number cell-autonomously and non-autonomously [40], [50]. Finally, expression of Jeb or dAlk transgenes in neuroendocrine IPCs using the dILP2-Gal4 driver did not modify pupal size. Thus, although both dAlk and dInr RTKs are involved in body size determination, their mechanisms and sites of action are distinct. This interpretation is consistent with results with the *C. elegans* Alk homolog Scd-2 shown to function in parallel with or converge with TGF-β signaling, but act independently of the Insulin cascade in dauer determination [51]. However, given that dInr and dAlk are members of the same subfamily of RTKs, a potential explanation for the lack of rescue of *dNf1* mutant homozygous larvae with systemic administration of 100 µM TAE684 (Figure 6C), may be off-target inhibition of dInr at the higher drug concentration [44].

Interestingly, S6K (dS6K) resides on a downstream branch of the dInr/PI(3)K signaling pathway and regulates cell size without impinging on cell number [41]. Although the dS6K loss-of-function phenotype resembles the dAlk gain-of-function and dNf1 loss-of-function phenotypes, its mode of action is cell-autonomous. However, it is still tempting to speculate that dAlk and dNf1 ultimately affect neuroendocrine signals that affect dS6K activity in peripheral tissues.

Increasing signaling through the cyclic AMP (cAMP)-dependent protein kinase A (PKA) pathway has been reported to suppress the size defect of *dNf1* mutants [30]. This among other findings, have led some to propose that dNf1 regulates Ras activity and cAMP levels independently [52], [53]. In contrast, an investigation of the cAMP/PKA sensitive *dNf1* mutant growth defect argued that aberrantly upregulated Ras/ERK signaling in Ras2-expressing larval neurons was its proximal cause [31]. Our results further support the latter explanation implicating a Ras/ERK signaling defect downstream of dAlk as the cause of size defects in *dNf1* mutants. Then, how could elevated cAMP/PKA signaling rescue decreased body size? Because neuroendocrine signals can activate the cAMP pathway [54], it is possible that defective dAlk/Ras/ERK signaling in *dNf1* mutants may lead to a neuroendocrine deficiency, which is restored by increasing cAMP/PKA signals.

### The role of dAlk in associative learning

In Drosophila, the dAlk/Jeb receptor-ligand pair has been shown to act in an antrerograde signaling pathway essential for assembly of the neuronal circuitry of the fly visual system [23]. However, loss of either Alk or Jeb did not appear to impair assembly of functional synapses with normal postsynaptic responses at the larval neuromuscular junction [24], indicating that they do not participate in CNS development. In agreement, pan-neuronal, spatially restricted attenuation or unregulated activation of Alk throughout development did not appear to yield gross structural defects in the adult brain (Figure 1 and not shown) or alter naïve behavioral responses to the stimuli used for conditioning (Table S1). Hence, it is unlikely that the learning phenotypes we describe are the result of developmental alterations in the CNS. In fact, dAlk seems to be acutely required for normal learning as limiting modulation of its activity to the adult CNS results in phenotypes on its own and also modifies the learning deficits of *dNf1* mutants. Moreover, the function of dAlk and dNf1 in associative learning is independent of body size as the learning reverted to normal by dAlk abrogation in the small-sized *dNf1* mutant homozygotes. Interestingly, the *C. elegans* Jeb homolog Hen-1 is required non-cell autonomously in the mature nervous system for sensory integration and associative learning [55]. Collectively then, these studies in *C. elegans*, mice [55], [56] and our data strongly support an evolutionary conserved role for Alk signaling in adult associative learning and memory.

Elevated dAlk/Jeb signaling outside the MBs impaired olfactory learning, while its abrogation increased learning efficiency. These are results are consistent with the enhanced performance in a hippocampus-dependent task described for Alk knockout mice [56]. We propose then, that Alk signaling normally functions to limit the strength of the CS/US associations, in effect providing a putative threshold required to be overcome for specific and efficient association of the stimuli. A GABAergic neuron outside the MBs, the Anterior Paired Lateral (APL), was recently reported to similarly suppress olfactory learning and its silencing yielded enhanced performance [57]. Interestingly, a decrease in presynaptic GABA release or abrogation of the GABA_A_ receptor, RDL in the post-synaptic mushroom body neurons [57], [58] resulted in enhanced learning. Whether *Ras2*-Gal4 is expressed in the APL neuron and dAlk also functions in this neuron to suppress learning are questions currently under investigation.

Interestingly, a recent study [59] suggested that the learning defects of Nf1^+/−^ mice are attributed to increased ERK-mediated phosphorylation of synapsin I in hippocampal inhibitory neurons and concomitant increase in GABA release. In accord, a GABA_A_ receptor antagonist enhanced learning in Nf1^+/−^ mice and controls, and reversed LTP defects in the mutants. Similarly, elimination of the dAlk-mediating inhibition in Drosophila Ras2-expressing neurons enhanced learning, potentially via attenuation of ERK phosphorylation. In support of this notion, we show that constitutive activation of ERK in adult Ras2-expressing neurons precipitates learning deficits (Figure S3D). Collectively, these results together with the reported learning deficits of Drosophila synapsin mutants [60], suggest that a mechanism similar to that proposed for vertebrates may also regulate Nf1-dependent learning in flies.

In mice, a decrease in Nf1 levels in heterozygous mutants increased Ras/ERK signaling and precipitated Long-Term Potentiation (LTP) and spatial learning deficits [61]. These deficits were reversed upon genetic or pharmacological inhibition of Ras signaling [61], [62]. Our own results demonstrate that dAlk inhibition reversed the impaired learning of *dNf1* mutants and since this is the first ‘kinase-active’ RTK shown to be involved in this process in flies [5], it provides independent support for Ras/ERK hyperactivation as causal of these learning defects. Then, how can the reported phenotypic reversal of *Nf1* learning deficits by expressing the PKA catalytic subunit throughout the fly [29] be explained? We hypothesize that the MBs are functionally downstream of the dAlk/dNf1 neurons and elevated PKA activity within the former could result in normal learning. Future work will focus on addressing the merits of these hypotheses regarding the mechanisms underlying the size and learning defects of *dNf1* mutants.

A recent report [28] suggested that *dNf1* mRNA is found in the mushroom bodies and in agreement, our own immunohistochemical results demonstrate that dNf1 is present within the mushroom body calyces (Figure 6, Figure S4). Protein synthesis-dependent memory defects in *Nf1* mutants were rescued upon MB-limited expression of the same full-length transgene as we used herein [28]. Since we did not examine memory deficits in our work, this complements our data and suggests a function for dNf1 within the MBs. In contrast, our data indicate that dNf1 expression in the MBs is not sufficient for learning/3 min memory (Figure 7G). Three common MB drivers (Figure 7D, 7E, 7F) including the most specific *MB247* and the most broadly expressed *OK107*-Gal4 [35], did not rescue learning in *Nf1* mutants by expressing dNf1. We suggest therefore that rescue described by Buchanan and Davis was mediated largely by *c739*-Gal4 transgene expression in neurons extrinsic to MBs where *Elav, Ras2* and *Alk(38)*-Gal4 are expressed, perhaps in combination with expression within MB-intrinsic neurons. The neuronal circuits where dNf1 and dAlk are required for normal learning are the subject of ongoing investigations.

### A potential therapeutic role for Alk

Our study identifies dAlk as the first RTK to functionally interact with Nf1 in Drosophila, raising the important question whether a similar functional relationship exists in mammals. Suggestive evidence argues that this may indeed be the case. Thus, Alk and NF1 extensively colocalize in the mammalian CNS during the same developmental periods [11], [26], [43]. Additionally, excess Alk expression or activation has been reported in astrocytomas, gliomas, neuroblastomas and pheochromocytomas, in which loss of *NF1* expression has also been found [11], [13]–[17], [63]. Based on our identification of Alk as a *bona-fide* RTK that initiates a Ras/ERK cascade regulated by Nf1, this suggests that Alk inhibition may rescue not only the phenotypes reported here, but also other symptoms that have been previously associated with Nf1 loss and ERK over-activation. It was recently reported that knockdown of *NF1* expression renders a neuroblastoma cell line resistant to retinoic acid-induced differentiation, and that NF1 deficient neuroblastoma tumors have a poor outcome [64]. Our results suggest that Alk inhibition may provide an intervention strategy in such cases. Finally, the findings reported here, combined with the lack of overt abnormalities in Alk knock-out mice [11], [16], [56], provide a rationale for further explorations of Alk as a potential therapeutic target in NF1.

## **Materials and Methods**

### Drosophila culture, strains, and genetics

Drosophila crosses were set up in standard wheat–flour–sugar food supplemented with soy flour and CaCl_2_, and cultured at 25°C and 50% humidity with a 12 h light/dark cycle. The *Alk^1^* and *Alk^9^*, *dNf1^E1^* and *dNf1^E2^* mutants have been described previously [22], [31]. Transgenic fly strains used in this work were: UAS*-Alk^WT^*, UAS*-Jeb*, UAS*-Alk^CA^*, UAS*-Alk^DN^* [20], [23], [33], UAS*-Alk^RNAi^* (Vienna Drosophila RNAi Center-11446), UAS-*Alk^RNAiKK^* (Vienna Drosophila RNAi Center-KK107083), UAS*-dNf1* [31], UAS*-rl^sem^* [45], UAS*-mCD8:: GFP* [65], Gal80^ts^ [34]. The MB-specific Gal80 (MBGal80), which drives expression predominantly in the MBs [47] was introduced into the UAS-*dNf1*,*dNf1^E1^* strain through standard genetic crosses. All strains were backcrossed into the Cantonised-*w^1118^* isogenic background for six generations. The legend to Table S2 gives the origin of all Gal4-driver lines used in this study.

### RTK expression profiling in the adult brain

Dissected adult *w^1118^* brains were submitted to Trizol-based RNA extraction followed by oligo-dT primed Reverse Transcription as described previously [66]. Amplification of cDNAs encoding Drosophila RTKs was achieved with the polymerase chain reaction (PCR) using a set of 21 distinct forward/reverse primers designed with Oligo v6.71 software (Molecular Biology Insights Inc.), using sequence information obtained from Flybase, Kinbase or NCBI GenBank. We probed for the following molecules that contain an RTK signature: dAlk, dTor, dRor, Nrk, sev, InR, btl, htl, Cad96Ca, Pvr, dRet, dnt, drl, Drl-2, Eph, Ddr, otk, Tie, CG3277, dEgfr and Wsck. All amplifications were performed in triplicate from two different pools of dissected brains for 24 and 32 cycles. RTKs whose cDNAs exhibited robust amplification at 24 cycles were selected for further investigation of their distribution in the adult brain using antibodies or reporter transposon insertions as available as a counter screen. RTKs with unequivocal presence in the MBs, or other spatial restriction in their distribution were selected for further characterization. Additional details and results from this screen will be presented elsewhere.

### Generation of Alk-Gal4 driver lines

The *Alk(38)*-Gal4 transgenic line was constructed by fusing a genomic region that includes the first (non-coding) exon and first intron of *dAlk*, to a cDNA encoding Gal4, followed by the genomic region downstream of the *dAlk* coding region encompassing the 3′UTR. Firstly, a clone corresponding to the presumed promoter region upstream of *dAlk*, was generated by PCR using genomic DNA and the primers ALK-FOR and ALK-REV (for primer sequences see below). These primers amplify a region extending from the first (non-coding) exon of the adjacent upstream gene, *gprs*, to 8 nt upstream of the ATG site of *Alk* (i.e. includes the first exon, first intron and part of the second exon of *dAlk*). Secondly, a Gal4 cDNA was amplified from the pChS vector using the Gal4-FOR and Gal4-REV primers. Finally, the region encoding the 3′UTR was amplified using ALK 3′UTR-FOR and ALK 3′UTR-REV. Each PCR product was TA-subcloned into pCR2.1 and sequenced.

The final Alk-Gal4 construct was made in a modified pCaSpeR1 vector containing the multiple cloning site of pBlueScript. Firstly, the upstream *dAlk* promoter was subcloned using Kpn I and Bam HI. Next, the Gal4 coding region was subcloned into the resulting construct with Bam HI and the orientation of the insert was verified. Finally, the *dAlk* 3′UTR was subcloned behind the Gal4 coding region using StuI and NotI.

ALK-FOR GGTACCCACACAGAAAGCAGAAG

ALK-REV GGATCCAGCTACACTTTTCACGTTT

GAL4-FOR GGATCCAACATGAAGCTACTGTCTTC

GAL4-REV AGGCCTTGCGGGGTTTTTCAGTATC

ALK 3′-FOR AGGCCTTACGCGGAGCGATACAAG

ALK 3′UTR-REV GCGGCCGCTGTGGAGCTCCTTTCTGGAG

Restriction sites are underlined

### Drug feeding

The selective Alk inhibitor TAE684 [44] was provided by Nathanael S. Gray, dissolved in DMSO and serial dilutions of stock solutions were prepared. For pupal measurements, the solution was subsequently mixed into 10 ml fly food to make the specific concentrations mentioned in the text. For behavioral experiments, the solution was mixed into 10 ml dead-dry-yeast paste and flies were fed for 48 h, and then transferred into normal fly-food vials 1 hour prior to conditioning. Green food dye (two drops per 10 ml) was added to monitor homogeneity and to ensure that larvae or adult flies were actually ingesting the food. No significant differences in the measurements of body size or learning performance were observed in flies fed without DMSO (no vehicle) or with DMSO alone (0 nM TAE684), confirming that DMSO showed no adverse effects.

### Morphometric analyses

Pupal size measurements were performed as described previously [31], [41]. ∼50 pupae of each genotype were digitally photographed using a video-equipped stereoscope (Zeiss stereoscope equipped with a Zeiss Axiocam CCD camera) and measured using ImageJ software (NIH Image 1.45). Pupae were then allowed to eclose, and scored for sex. Measurements for ∼20 female pupae were used to calculate average size, standard deviations, and statistical significance. To allow for slight variations in experimental conditions, all controls were included in each experiment. For cell growth analyses, ∼20 wings of 20 different female flies of each genotype were dissected, placed on slides and digitally photographed under the same magnification. Images were captured as described above. ImageJ software was used to measure wing-blade surfaces viewed at 40X magnification. The size of intervein cells was obtained by initially counting the number of hairs in a rectangle of 0.02 mm^2^ (cell density). In order to maintain fair morphological comparisons, the location of the rectangle was defined using wing vein landmarks (between veins 3 and 4 of the dorsal wing-blade, up to the posterior cross vein). The reciprocal value of the cell density gives the cell size. The approximate number of cells in the whole wing was calculated by multiplying the wing surface by the cell density. Similar results were obtained with male flies. Experiments were replicated at least once with flies from different crosses (biological replicates).

### Behavioral analyses and conditioning

Behavioral tests were performed under dim red light at 23°C–25°C and 70%–78% humidity. All animals were 2–6 days old, collected under light CO_2_ anesthesia one day prior to testing, and kept in food vials in groups of 50–70 at 23°C–25°C or 18°C as appropriate for strains with Gal80^ts^ temporal restriction of transgene expression. They were transferred to fresh vials 1–1.5 hours before testing. Olfactory learning and memory in the negatively reinforced paradigm coupling aversive odors as conditioned stimuli (CS+ and CS-) with the electric shock unconditioned stimulus (US) [67] was used to assess learning and memory. The aversive odors used were benzaldehyde (BNZ) and 3-octanol (OCT). For training, ∼50 flies were placed into a tube lined with an electrifiable grid and presented with air (500 ml/min) for 15 s, the shock-associated odor carried in the air current for 1 min concomitant with 1.25 second shocks at 90 V delivered every 5 s. This was followed by delivery of air for 30 s, the control odor in the air current for 1 min, and air again for 30 s. The timing of stimulus delivery was kept proportional to that for the full 12 CS/US pairing protocol, such that 3 shocks were delivered in 15 seconds of continuous CS+ presentation, 6 pairings within 30 seconds and so on. Two groups of animals of the same genotype were trained simultaneously, one to avoid BNZ, the other OCT, while the complementary odorant was used as the respective control. The animals were transferred to a T-maze apparatus immediately and allowed to choose between the two odors converging in the middle for 100 seconds. Since the time between testing and the coupling of the conditioned with the unconditioned stimulus is 3 min, the initial performance assessment is that of 3 min memory, which we refer to as learning. Performance was measured by calculating an index (PI), as the fraction of flies that avoided the shock-associated odor minus the fraction that avoided the control odor reflected learning due to one of the conditioning stimuli and represented half of the performance index. One performance index was calculated as the average of the half-learning indexes for each of the two groups of animals trained to complementary conditioning stimuli and ranges from 100 to 0, for perfect to no learning, respectively. All behavioral experiments were carried out in a balanced design, where all genotypes involved in an experiment were tested per day. The experimenter was blind to the genotype. Behavioral experiments were replicated at least once with flies from different crosses and a different time period (biological replicates). For experiments using the TARGET system (flies bearing Gal80^ts^), all animals were raised at 18°C until adulthood and UAS*-dAlk* transgenes were induced maximally by placing 3–5-day old flies at 30°C for 48 h. The animals were kept at the training temperature (25°C) for 30 min before training.

To assess olfactory avoidance, naive animals were given 100 seconds to choose between one of the odors and air. The airflow in both arms of the maze was kept constant and equal to that used for testing conditioned animals. Avoidance to both odors was tested simultaneously for each strain and all strains used were tested in a given session. Avoidance is represented by a performance index, which is calculated as the fraction of flies that avoid the odorant minus the fraction of flies that do not. To assess the avoidance of animals to electric shock, the arms of the T-maze were lined with electrifiable grids. Naive flies were placed at the choice point and given 100 seconds to choose between an electrified and an inert grid. Throughout the choice period, 1.25 s shocks at 90 V were delivered to one arm and air was passed through both arms at the standard flow rate. Avoidance is measured by a performance index calculated as the fraction of flies that avoid the electrified grid minus the fraction of flies that do not. Again, all strains were tested in a given session.

### Immunohistochemical analysis

For paraffin sections (Figure 1A), wild type animals were fixed in Carnoy's fixative (60% ethanol, 30% chloroform, 10% acetic acid) for 4 hr at room temperature, treated with methylbenzoate for 12 hr, and embedded in paraffin. 6 µm sections were obtained, deparaffinized in xylene baths, rehydrated through 100%–30% ethanol series, blocked for 1.5 h in 10% normal goat serum in PBHT [0.02 M NaPO_4_, 0.5 M NaCl, 0.2% Triton X-100 (pH 7.4)] and challenged with the rabbit anti-dAlk antibody (1∶1000) in blocking solution (5% normal goat serum in PBHT) overnight at 4°C. The sections were washed in PBHT, and a 1∶400 dilution of the secondary antibody (Vector Labs) in blocking solution was applied at room temperature for 3 hr. Slides were washed in PBHT and exposed to HRP conjugated to streptavidin at a dilution of 1∶400 in PBHT. After a final PBHT wash, the HRP was reacted with a substrate solution of 1 mg/ml diaminobenzidine and 0.03% H_2_O_2_ in PBHT. The unreacted substrate was washed away with water and the slides were mounted with Glycergel (DAKO).

Whole-mount larval CNS and adult brains were dissected in cold PBS, fixed in 4% paraformaldehyde for 20 min, and permeabilized using 0.1% Triton X-100 in PBS. The primary antibodies were used as follows: rabbit anti-dAlk (1∶1,000) [33], and mouse anti-Nf1 (DNF1-21) (1∶5) [30]. The following secondary antibodies were used: Goat anti-mouse, anti-rabbit conjugated with Alexa-Fluor secondary antibodies (1∶400, all from Molecular Probes). Confocal laser microscopy was performed using a Leica TCS SP5 Confocal system equipped with the Leica LAS AF image acquisition analysis software suite. Images were processed using ImageJ 1.45 (NIH, Bethesda) software.

### Western blot analysis

For detection of dpERK and total ERK levels, five adult heads or five larval CNS were homogenized in Laemmli buffer supplemented with protease and phosphatase inhibitors. Extract equivalent to one adult head or one larval CNS was loaded per lane and the primary antibodies were used at 1∶2,000 for mouse anti-pERK, (Sigma), and 1∶2,000 for rabbit anti-ERK (Cell Signaling). Mouse anti-tubulin (Developmental Studies Hybridoma Bank) at 1∶2,500 was used as an internal loading control. Four independent experiments were scanned, the band intensities were determined using ImageQuant 5.0 (Molecular Dynamics) and used to calculate ratios of dpERK/ERK.

### Statistical analysis

Untransformed (raw) data were analyzed parametrically with the JMP 7.1 statistical software package (SAS Institute Inc., Cary, NC) as described before [9]. Following initial ANOVA, planned multiple comparisons were performed, using α = 0.05. The level of significance was adjusted for the experimentwise error rate. Detailed results of all planned comparisons mentioned in the figure legends are shown in Table S3. Data are shown as mean ± S.E.M.

## Supporting Information

Figure S1 Neuroanatomic characterization of the *Alk(38)*-Gal4 and *Ras2*-Gal4 expression pattern. (A) Confocal images obtained from 3^rd^ instar larval CNS (1**–**3) acquired at two different optical sections. They present extensive colocalization between *Alk(38)*-Gal4-driven membrane-GFP (mGFP) expression and endogenous dAlk protein, revealing the specificity of this novel driver. Note the extensive overlap between mGFP and dAlk expression in the regions of the ventral ganglion and the central brain, in particular in larval calyces (white arrows). (B) Confocal images of a whole-mount adult central brain acquired at three different optical sections (1**–**3). They illustrate the extensive colocalization (white) between membrane-GFP and endogenous dAlk proteins, revealing the specificity of the *Alk(38)*-Gal4 driver in the adult brain. Note the endogenous dAlk labeling in calyces, but not in the lobes (arrows, 3 versus 1**–**2), indicating preferential targeting of dAlk in mushroom body dendrites. The *Alk(38)*-Gal4-driven membrane-GFP additionally labels axonal structures (green arrows, 1**–**2). dAlk protein is expressed in Ras2-expressing cells in the larval (C) and adult (D) CNS. (C) Confocal imaging of third instar larval CNS. Inset: higher magnification of the hatched boxes, showing colocalization of dAlk as visualized with anti-Alk immunofluorescence, and *Ras2*-Gal4-driven membrane-GFP in single neurons. (D1-3) Confocal imaging of three different optical z-sections in adult brain, showing co-localization of dAlk as visualized with anti-Alk immunofluorescence and *Ras2*- Gal4-driven membrane-GFP. Arrows indicate the substantial accumulation of dAlk protein in the mushroom body calyces (3). Bars = 50 µm.
(TIF)

Figure S2 Size alterations of *dNf1*-null and flies pan-neuronally expressing *dAlk* transgenes. Adult flies of the indicated genotypes exhibit clear size differences but are normally patterned and of proportionally altered size. Adult female flies homozygous for *Nf1^E1^* and *Nf1^E2^ null* alleles (upper row) or expressing pan-neuronally (*Elav*-Gal4) the indicated UAS-*dAlk* or UAS-*Jeb* transgenes (lower row) are shown. Wild-type *w^1118^* controls are also shown for comparison.
(TIF)

Figure S3 Cell size but not cell number increases upon rescuing the size defects of *Nf1^E2^* homozygotes by dAlk reduction. (A) Amelioration of *Nf1^E2^* homozygous mutant size deficits by the heterozygous *Alk^1^-null* and *Alk^9^-dead-kinase* mutant alleles is attributed specifically to increase of cell size and not of cell proliferation. ANOVA indicated significant effects of genotype on wing size (F_(5,102)_ = 197.85, p<0.0001, n>16) and cell size (F_(5,102)_ = 172.80, p<0.0001, n>16), but not on total cell number (F_(5,102)_ = 0.57, p<0.72, n>16). Planned pairwise comparisons between *Nf1^E2^* and *Alk* mutants in *Nf1^E2^* mutant background showed significant differences on wing size and cell size indicating rescue (p<0.0001 for all comparisons). (B) Activation of dAlk signaling in neurons, neuroendocrine and Ras2-expressing cells enhances the size deficits exhibited by *Nf1^E2^*/+ mutants. Over-activation of dAlk signaling further decreases the reduced size of *Nf1^E2^/+*, but to a lower extent, which allows larvae to survive and thus reach pupal stage. Note that inhibition of dAlk signaling in heterozygous *Nf1^E2^/+* mutants fully restored size deficits. ANOVA indicated significant effects of genotype for all drivers tested (*Elav*- Gal4: F(4,259) = 677.12, p<0.0001,*386Y*-Gal4: F(5,263) = 909.92, p<0.0001, *Ras2*-Gal4: F(4,259) = 522.69, p<0.0001, *OK107*- Gal4: F(5,301) = 19.75, p<0.0001). Planned comparisons showed significant differences between heterozygous *Nf1^E2^* flies and flies over-expressing dAlk transgenes (p<0.0001 for *Elav*-Gal4, *386Y*-Gal4 and *Ras2*-Gal4 for all genotypes). No significant differences were observed between heterozygous *Nf1^E2^* flies and flies over-expressing dAlk transgenes using the *OK107*-Gal4 driver (p>0.1 for all genotypes). (C) ERK activity in Ras2-expressing cells controls organism size. Expression of an activated form of *rolled/*ERK (UAS*-rl^sem^*) in larval Ras2-expressing cells results in pupal size reduction, through reduction of cell size and not cell proliferation. t-tests between driver heterozygote control flies and flies expressing UAS*-rl^sem^* showed highly significant effects on pupal size (t_(55)_ = 18.68, p<0.0001, n>25). Measurements of wing hairs revealed highly significant differences on cell size (t_(17)_ = 10.81, p<0.0001, n>8) but not cell number (t_(17)_ = 0.01, p>0.98, n>8). Error bars denote S.E.M. (D) ERK activity in Ras2-expressing cells results in learning deficits in adult flies. This deficit is clearly produced by transgene expression as it is absent in flies grown at the restrictive temperature (‘Un.’: Uninduced). ANOVA indicated significant effects of genotype (F_(2,19)_ = 6.25, p<0.0028, n>6). Post-hoc planned comparisons showed significant differences between adult flies expressing UAS*-rl^sem^* in Ras2-expressing cells (‘In.’) and heterozygous driver control flies (p<0.004) or flies tested in the restrictive temperature (‘Un.’) (p<0.01). Error bars denote S.E.M.
(TIF)

Figure S4 Control staining for a-dAlk antibody specificity and Nf1 expression pattern. (A) 6 µm frontal paraffin sections were stained without addition of primary anti-dAlk antibody. No immunoreactivity above background levels was detected at the level of calyces and protocerebral bridge (1) or at the level of the MB pedunculus and fan-shaped body (2). (B) Representative optical sections from brains of adult *w^1118^* flies stained with the anti-dNf1 monoclonal antibody. (1) dNf1 protein clearly accumulates within the dendrites *(calyces, ca)* and cell bodies *(cb)* of mushroom body neurons, as well as in the protocerebral bridge *(pb)*, the medial bundle *(mb)* (3) and the sub-oesophageal ganglia *(sog)* (3). It is also widely expressed in the neuropil and distinct peripheral glomeruli of the antennal lobes (al) (4), barely above general background staining. However, Nf1 staining appears equivalent to background levels in the axons of mushroom body neurons (pedunculus, p and α, β and γ lobes) (2,3). Confocal images were acquired at the same section levels and using the same settings. They were then converted to grayscale and inverted. Scale bars = 50 µm.
(TIF)

Figure S5 Learning deficits of *Nf1* null mutants and their rescue upon abrogation of endogenous dAlk. (A) *Nf1^E1^ and Nf1^E2^ homozygous* null mutants exhibit significant learning defects. ANOVA indicated significant effects of genotype (F_(3,29)_ = 35.90, p<0.0001, n>7). Subsequent planned comparisons between *Nf1^E1^, Nf1^E2^* or *Nf1^E1/E2^* null flies and *w^1118^* controls (p<0.0001 for all comparisons). Null *Nf1^E1/E2^* heteroallelics show equivalent performance to *Nf1^E2^* homozygotes used in the study (p = 0.51). (B) *Nf1^E1/+^ and Nf1^E2^/+* exhibit significant learning defects under limited CS/US associations during training. Reduced number of CS/US associations (3) revealed learning deficits in *Nf1^E1/+^ and Nf1^E2/+^ heterozygous* null mutants (F_(2,17)_ = 4.63, p<0.02, n>5, p<0.01 for *Nf1^E1/+^ and Nf1^E2/+^* compared to control) that were not observed with 6CS/US, which led to performance equal with *w^1118^* controls (F_(2,19)_ = 1.67, p>0.21, n>6). (C) Rescue of *Nf1^E2^* learning deficits using a second independent UAS*-Alk^RNAi^* transgene. Pan-neuronal expression of a second UAS*-Alk^RNAi^* transgene (UAS*-Alk^RNAiKK^*: VDRC KK107083) in adult *Nf1^E2^* flies rescues learning deficits. ANOVA indicated significant effects of genotype (F_(2,19)_ = 8.13, p<0.0037, n>6 for all genotypes). Planned pairwise comparisons indicated significant differences between *Nf1^E2^* mutant flies harboring the *Elav*-Gal4;Gal80^ts^ driver and heterozygous driver controls or *Nf1^E2^* mutant flies with down-regulated dAlk signaling in neurons (p<0.001 and p<0.005 for *Elav*-Gal4;Gal80^ts^ and Elav-Gal4;UAS*-Alk^RNAiKK^*/+;Gal80^ts^, E2 respectively). Expression in Ras2-expressing cells of a second UAS*-Alk^RNAi^* transgene (UAS*-Alk^RNAiKK^*: VDRC KK107083) in adult *Nf1^E2^* flies rescues learning deficits. ANOVA indicated significant effects of genotype (F_(2,37)_ = 29.54, p<0.0001, n>11 for all genotypes). Planned pairwise comparisons indicated significant differences between *Nf1^E2^* mutant flies harboring the *Ras2*-Gal4;Gal80^ts^ driver and heterozygous driver controls or mutant flies with down-regulated dAlk signaling in Ras2-expressing cells (p<0.0001 for all comparisons).
(TIF)

Table S1 Task relevant sensory behaviors. Avoidance of the aversive odor stimuli (CS) and electric shock (US) is shown for all relevant strains (n>6 for all odor avoidance and n>4 for all shock avoidance experiments). Values in rows indicate the mean Performance Index (PI) ± S.E.M. of flies avoiding the corresponding stimulus (Benzaldehyde, Octanol or Electric Shock). ANOVA values are shown below each group tested. Avoidance of strains in each group was compared to that of their proper heterozygous driver control. Values in bold writing indicate statistical significant differences from control strain. All strains were not tested simultaneously, although all strains within a group were. Thus, statistical analyses for performance differences were performed exclusively within each group.
(DOC)

Table S2 Pupal size alterations result from tissue-specific expression of UAS*-dAlk* and UAS-*Jeb* transgenes. The numbers in the ‘*Ref.*’ column refer to the reference list given below the table. ‘*Expression in the 3^rd^ instar larval Nervous System*’ briefly describes the main neural tissues in which the Gal4-drivers are expressed. Details come either from already published data or determined by immunohistochemistry we performed on dissected 3^rd^ instar larvae nervous system obtained from crosses of Gal4-drivers to UAS-*mCD8::GFP* flies. Data on the ‘*Effect of UAS-dAlk transgenes on pupal size*’ were obtained from crosses of Gal4-drivers to UAS-*dAlk^WT^,* UAS-*Jeb,* UAS-*dAlk^CA^,* UAS-*dAlk^DN^,* UAS-*dAlk^RNAi^* strains. Each cross was set up using an equal number of females per vial and at least 15 pupae of both sexes were measured for each Gal4-driver (n = 15**–**50). Pupal size measurements were performed as described in the ‘Materials and Methods’ section. The resulting effect on pupal size is qualitatively represented by upward (↑ for increase), or downward (↓ for reduced) arrows for those Gal4-drivers that significantly altered pupal size, and by horizontal arrrows (→) for those that do not significantly alter size (Dunnett's test, p<0.001). Crosses (†) represent larval lethality, and thus not measurable at the pupal stage. Abbreviations: AL: Antenna lobe; CB: central brain; CCAP: Crustacean cardioactive peptide; CNS: central nervous system; DPM: Dorsal paired medial neurons; MB: Mushroom Body neurons; MBextr: extrinsic mushroom bodies neurons (presynaptic neurons that project to MBs); MBintr: intrinsic mushroom bodies neurons (postsynaptic neurons constituent of mushroom bodies); PN; Projection neurons; RG: ring gland; SG: salivary glands; VG: ventral ganglion.
(DOC)

Table S3 Results of planned comparisons for Figure 2, Figure 3, Figure 6, and Figure 7 in the main article. The scores of all genotypes were compared per group (each experimental group separated by an empty row) with the relevant genotype listed first (indicated by #). Significant differences are denoted by the star sign. The level of significance was adjusted for the experimentwise error rate.
(DOC)
