# Supplementary figures and images for: The Receptor Tyrosine Kinase Alk Controls Neurofibromin Functions in Drosophila Growth and Learning
Source: PLoS Genet. 2011 Sep 15;7(9):e1002281. doi: 10.1371/journal.pgen.1002281 (PMC3174217; doi:10.1371/journal.pgen.1002281)

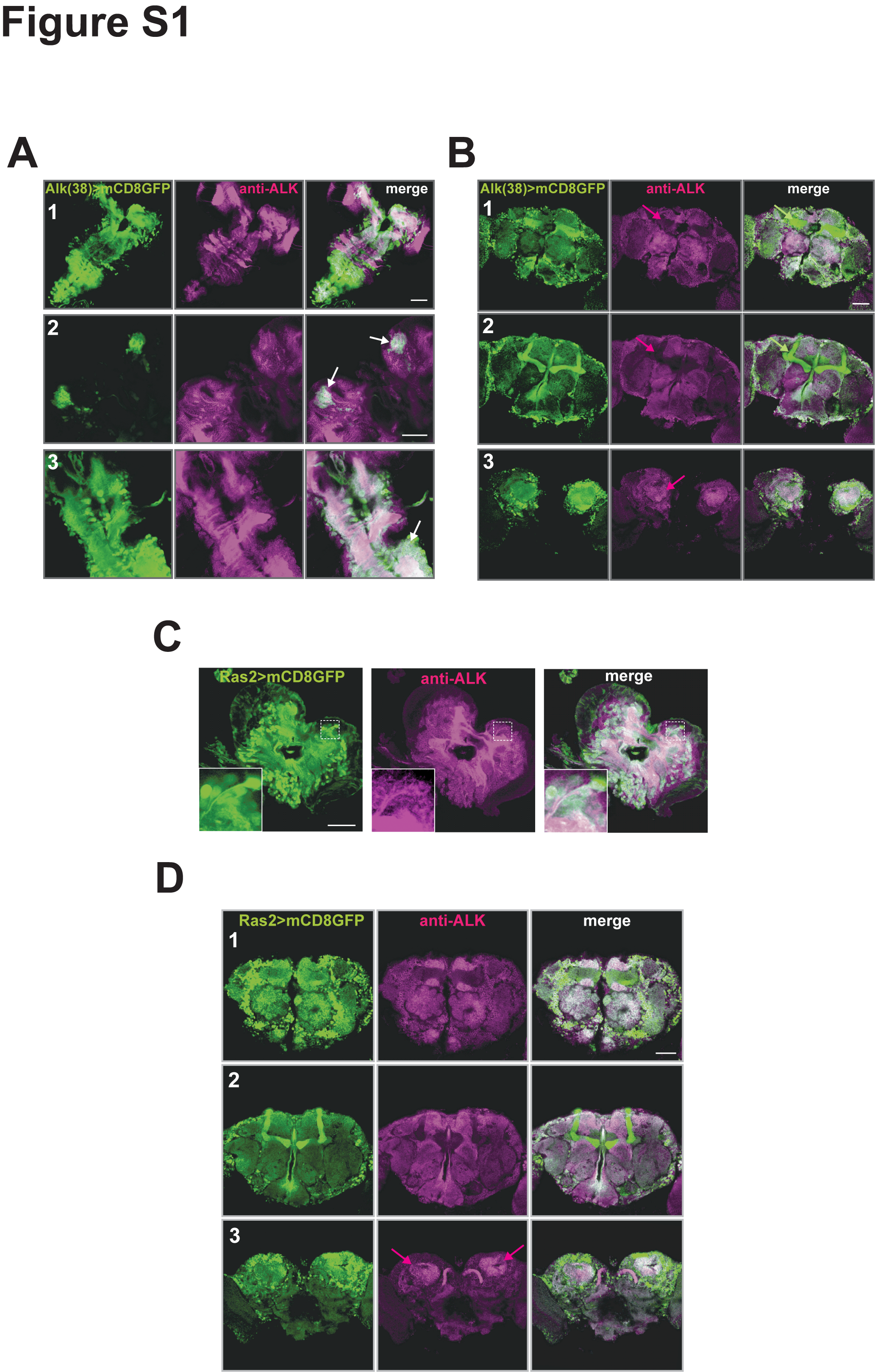

Supplement: Figure S1 — Neuroanatomic characterization of the Alk(38)-Gal4 and Ras2-Gal4 expression pattern. (A) Confocal images obtained from 3rd instar larval CNS (1–3) acquired at two different optical sections. They present extensive colocalization between Alk(38)-Gal4-driven membrane-GFP (mGFP) expression and endogenous dAlk protein, revealing the specificity of this novel driver. Note the extensive overlap between mGFP and dAlk expression in the regions of the ventral ganglion and the central brain, in particular in larval calyces (white arrows). (B) Confocal images of a whole-mount adult central brain acquired at three different optical sections (1–3). They illustrate the extensive colocalization (white) between membrane-GFP and endogenous dAlk proteins, revealing the specificity of the Alk(38)-Gal4 driver in the adult brain. Note the endogenous dAlk labeling in calyces, but not in the lobes (arrows, 3 versus 1–2), indicating preferential targeting of dAlk in mushroom body dendrites. The Alk(38)-Gal4-driven membrane-GFP additionally labels axonal structures (green arrows, 1–2). dAlk protein is expressed in Ras2-expressing cells in the larval (C) and adult (D) CNS. (C) Confocal imaging of third instar larval CNS. Inset: higher magnification of the hatched boxes, showing colocalization of dAlk as visualized with anti-Alk immunofluorescence, and Ras2-Gal4-driven membrane-GFP in single neurons. (D1-3) Confocal imaging of three different optical z-sections in adult brain, showing co-localization of dAlk as visualized with anti-Alk immunofluorescence and Ras2- Gal4-driven membrane-GFP. Arrows indicate the substantial accumulation of dAlk protein in the mushroom body calyces (3). Bars = 50 µm. (TIF) [file pgen.1002281.s001.tif]

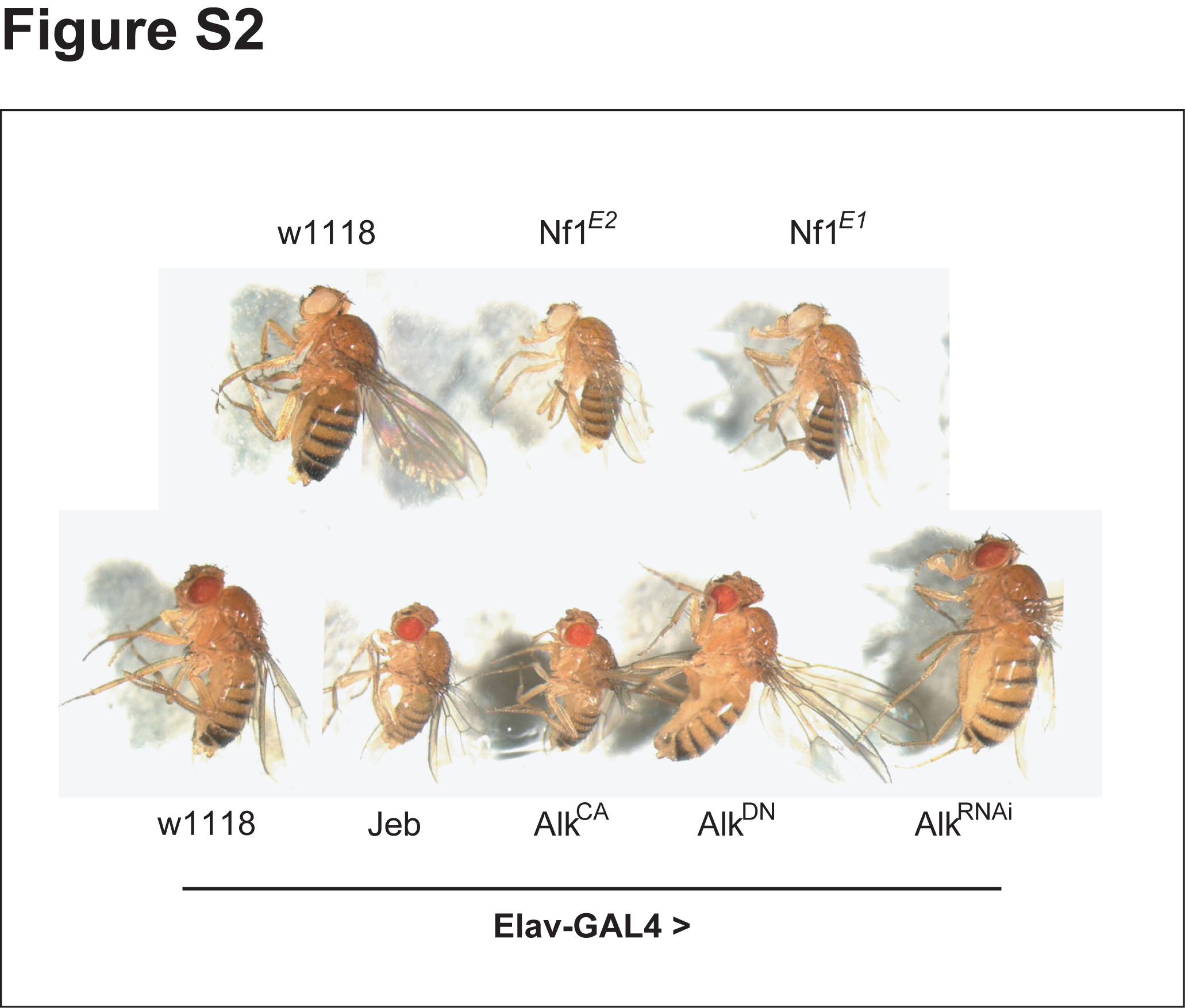

Supplement: Figure S2 — Size alterations of dNf1-null and flies pan-neuronally expressing dAlk transgenes. Adult flies of the indicated genotypes exhibit clear size differences but are normally patterned and of proportionally altered size. Adult female flies homozygous for Nf1E1 and Nf1E2 null alleles (upper row) or expressing pan-neuronally (Elav-Gal4) the indicated UAS-dAlk or UAS-Jeb transgenes (lower row) are shown. Wild-type w1118 controls are also shown for comparison. (TIF) [file pgen.1002281.s002.tif]

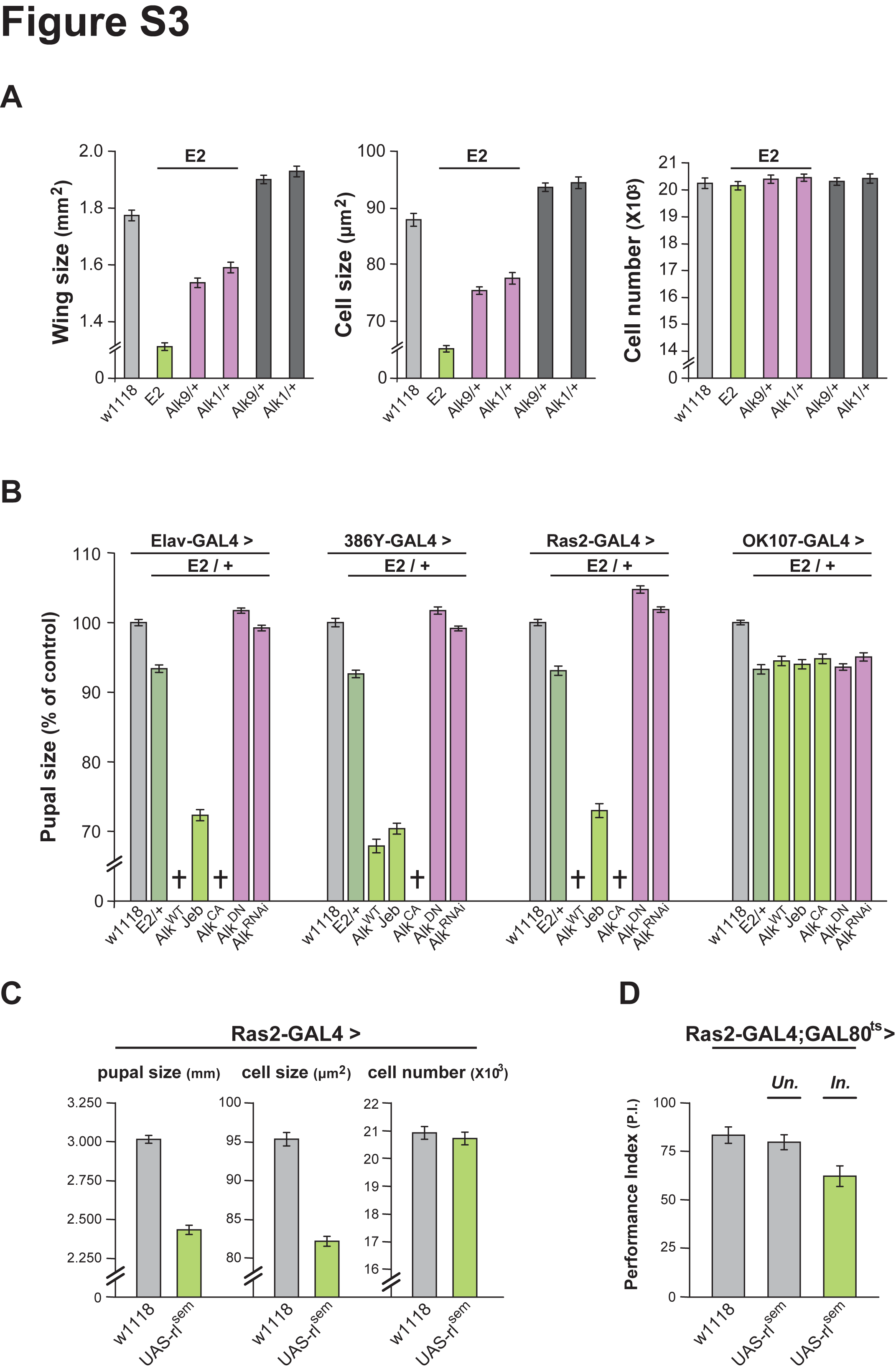

Supplement: Figure S3 — Cell size but not cell number increases upon rescuing the size defects of Nf1E2 homozygotes by dAlk reduction. (A) Amelioration of Nf1E2 homozygous mutant size deficits by the heterozygous Alk1-null and Alk9-dead-kinase mutant alleles is attributed specifically to increase of cell size and not of cell proliferation. ANOVA indicated significant effects of genotype on wing size (F(5,102) = 197.85, p<0.0001, n>16) and cell size (F(5,102) = 172.80, p<0.0001, n>16), but not on total cell number (F(5,102) = 0.57, p<0.72, n>16). Planned pairwise comparisons between Nf1E2 and Alk mutants in Nf1E2 mutant background showed significant differences on wing size and cell size indicating rescue (p<0.0001 for all comparisons). (B) Activation of dAlk signaling in neurons, neuroendocrine and Ras2-expressing cells enhances the size deficits exhibited by Nf1E2/+ mutants. Over-activation of dAlk signaling further decreases the reduced size of Nf1E2/+, but to a lower extent, which allows larvae to survive and thus reach pupal stage. Note that inhibition of dAlk signaling in heterozygous Nf1E2/+ mutants fully restored size deficits. ANOVA indicated significant effects of genotype for all drivers tested (Elav- Gal4: F(4,259) = 677.12, p<0.0001,386Y-Gal4: F(5,263) = 909.92, p<0.0001, Ras2-Gal4: F(4,259) = 522.69, p<0.0001, OK107- Gal4: F(5,301) = 19.75, p<0.0001). Planned comparisons showed significant differences between heterozygous Nf1E2 flies and flies over-expressing dAlk transgenes (p<0.0001 for Elav-Gal4, 386Y-Gal4 and Ras2-Gal4 for all genotypes). No significant differences were observed between heterozygous Nf1E2 flies and flies over-expressing dAlk transgenes using the OK107-Gal4 driver (p>0.1 for all genotypes). (C) ERK activity in Ras2-expressing cells controls organism size. Expression of an activated form of rolled/ERK (UAS-rlsem) in larval Ras2-expressing cells results in pupal size reduction, through reduction of cell size and not cell proliferation. t-tests between driver [file pgen.1002281.s003.tif]

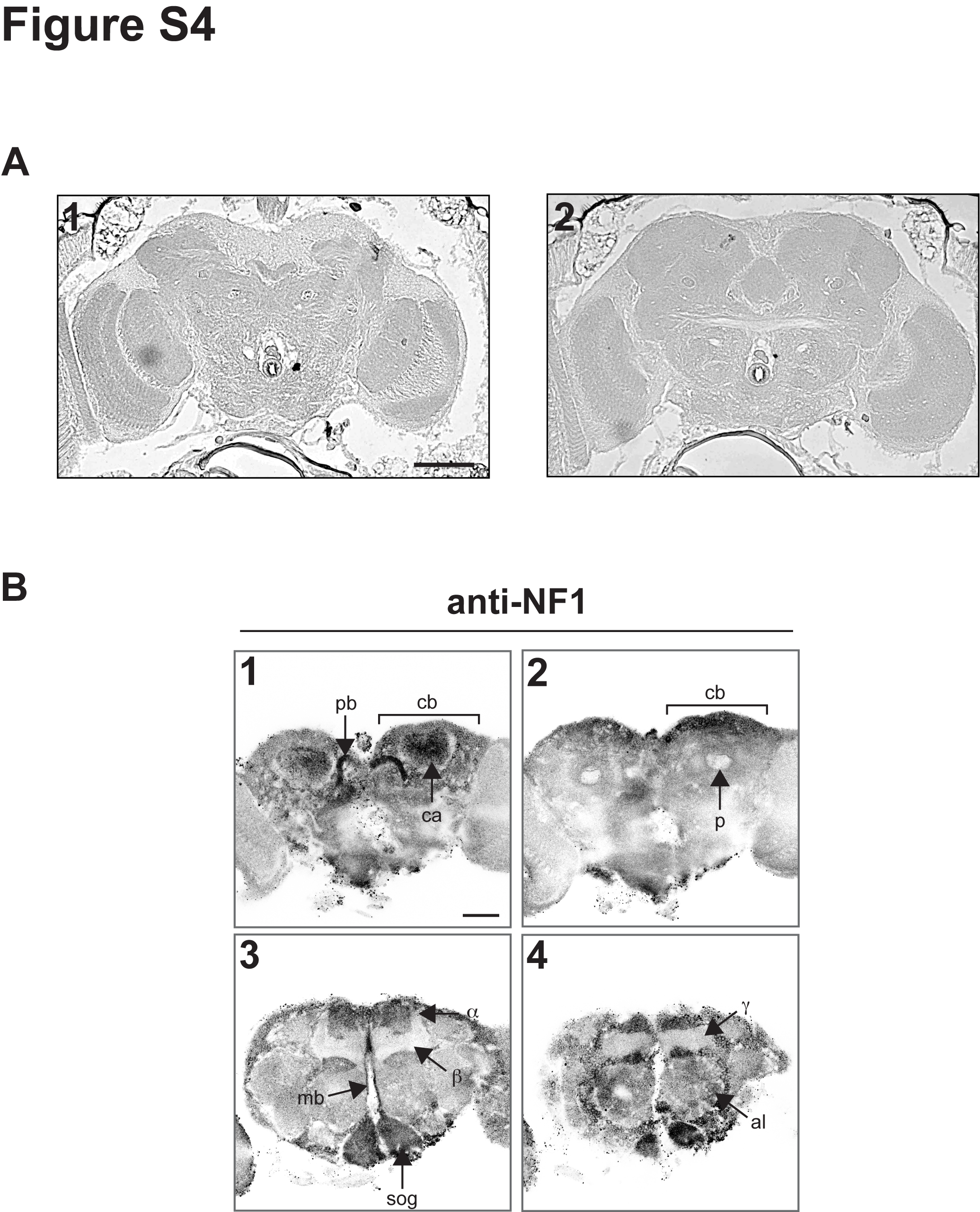

Supplement: Figure S4 — Control staining for a-dAlk antibody specificity and Nf1 expression pattern. (A) 6 µm frontal paraffin sections were stained without addition of primary anti-dAlk antibody. No immunoreactivity above background levels was detected at the level of calyces and protocerebral bridge (1) or at the level of the MB pedunculus and fan-shaped body (2). (B) Representative optical sections from brains of adult w1118 flies stained with the anti-dNf1 monoclonal antibody. (1) dNf1 protein clearly accumulates within the dendrites (calyces, ca) and cell bodies (cb) of mushroom body neurons, as well as in the protocerebral bridge (pb), the medial bundle (mb) (3) and the sub-oesophageal ganglia (sog) (3). It is also widely expressed in the neuropil and distinct peripheral glomeruli of the antennal lobes (al) (4), barely above general background staining. However, Nf1 staining appears equivalent to background levels in the axons of mushroom body neurons (pedunculus, p and α, β and γ lobes) (2,3). Confocal images were acquired at the same section levels and using the same settings. They were then converted to grayscale and inverted. Scale bars = 50 µm. (TIF) [file pgen.1002281.s004.tif]

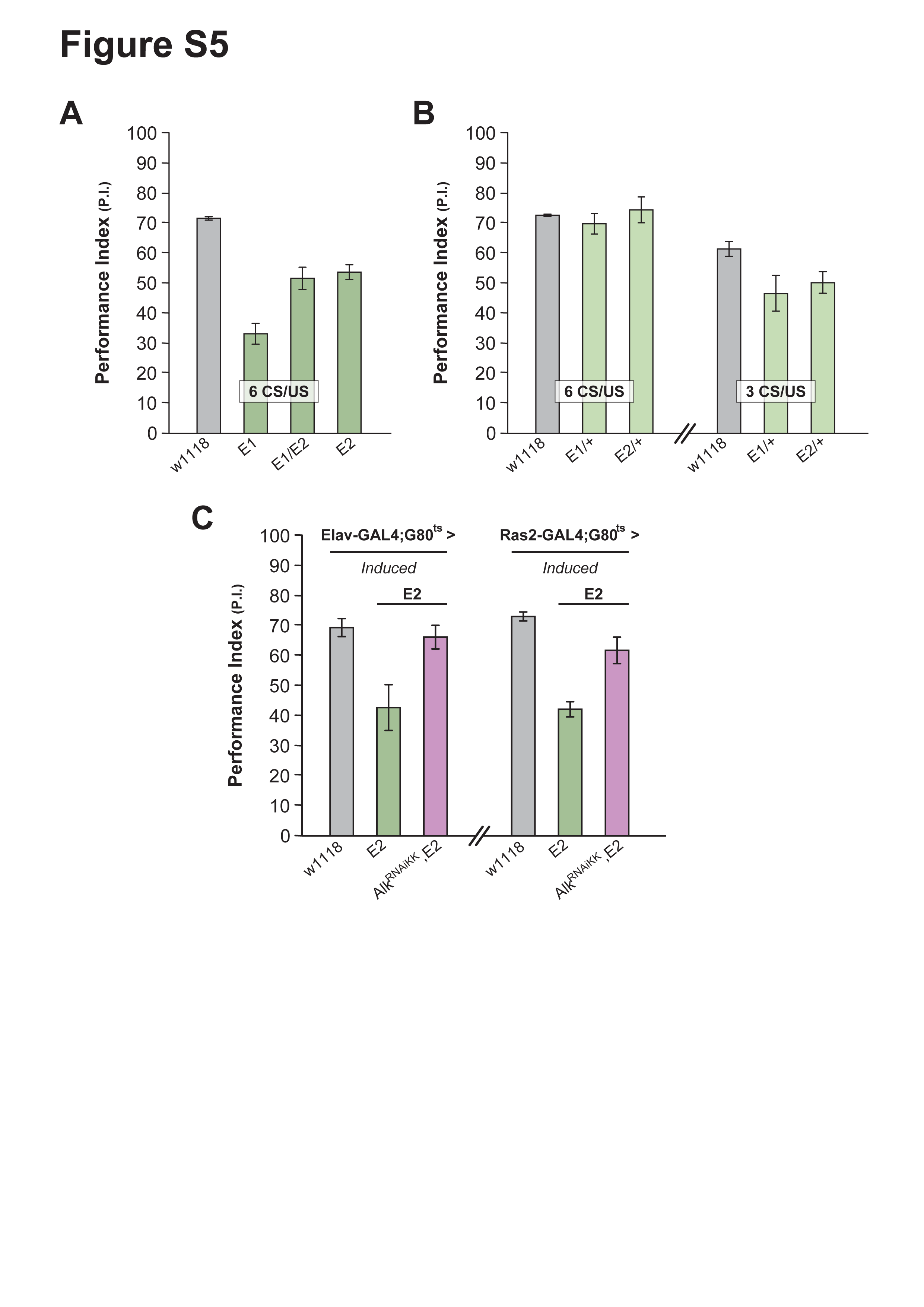

Supplement: Figure S5 — Learning deficits of Nf1 null mutants and their rescue upon abrogation of endogenous dAlk. (A) Nf1E1 and Nf1E2 homozygous null mutants exhibit significant learning defects. ANOVA indicated significant effects of genotype (F(3,29) = 35.90, p<0.0001, n>7). Subsequent planned comparisons between Nf1E1, Nf1E2 or Nf1E1/E2 null flies and w1118 controls (p<0.0001 for all comparisons). Null Nf1E1/E2 heteroallelics show equivalent performance to Nf1E2 homozygotes used in the study (p = 0.51). (B) Nf1E1/+ and Nf1E2/+ exhibit significant learning defects under limited CS/US associations during training. Reduced number of CS/US associations (3) revealed learning deficits in Nf1E1/+ and Nf1E2/+ heterozygous null mutants (F(2,17) = 4.63, p<0.02, n>5, p<0.01 for Nf1E1/+ and Nf1E2/+ compared to control) that were not observed with 6CS/US, which led to performance equal with w1118 controls (F(2,19) = 1.67, p>0.21, n>6). (C) Rescue of Nf1E2 learning deficits using a second independent UAS-AlkRNAi transgene. Pan-neuronal expression of a second UAS-AlkRNAi transgene (UAS-AlkRNAiKK: VDRC KK107083) in adult Nf1E2 flies rescues learning deficits. ANOVA indicated significant effects of genotype (F(2,19) = 8.13, p<0.0037, n>6 for all genotypes). Planned pairwise comparisons indicated significant differences between Nf1E2 mutant flies harboring the Elav-Gal4;Gal80ts driver and heterozygous driver controls or Nf1E2 mutant flies with down-regulated dAlk signaling in neurons (p<0.001 and p<0.005 for Elav-Gal4;Gal80ts and Elav-Gal4;UAS-AlkRNAiKK/+;Gal80ts, E2 respectively). Expression in Ras2-expressing cells of a second UAS-AlkRNAi transgene (UAS-AlkRNAiKK: VDRC KK107083) in adult Nf1E2 flies rescues learning deficits. ANOVA indicated significant effects of genotype (F(2,37) = 29.54, p<0.0001, n>11 for all genotypes). Planned pairwise comparisons indicated significant differences between Nf1E2 mutant flies harboring the Ras2-Gal4;Gal80ts driver and heterozygous driver controls or mutant flies w [file pgen.1002281.s005.tif]
