# Supplementary material for: The Receptor Tyrosine Kinase Alk Controls Neurofibromin Functions in Drosophila Growth and Learning
Source: PLoS Genet. 2011 Sep 15;7(9):e1002281. doi: 10.1371/journal.pgen.1002281 (PMC3174217; doi:10.1371/journal.pgen.1002281)
Supplement: Table S1 — Task relevant sensory behaviors. Avoidance of the aversive odor stimuli (CS) and electric shock (US) is shown for all relevant strains (n>6 for all odor avoidance and n>4 for all shock avoidance experiments). Values in rows indicate the mean Performance Index (PI) ± S.E.M. of flies avoiding the corresponding stimulus (Benzaldehyde, Octanol or Electric Shock). ANOVA values are shown below each group tested. Avoidance of strains in each group was compared to that of their proper heterozygous driver control. Values in bold writing indicate statistical significant differences from control strain. All strains were not tested simultaneously, although all strains within a group were. Thus, statistical analyses for performance differences were performed exclusively within each group. (DOC) [file pgen.1002281.s006.doc]

| **GENOTYPE** | **Benzaldehyde** | | | **Octanol** | | **Electric Shock** | |
| --- | --- | --- | --- | --- | --- | --- | --- |
|  | **PI** | | **+ S.E.M.** | **PI** | **+ S.E.M.** | **PI** | **+ S.E.M.** |
| Elav-GAL4/+;GAL80^ts^/+ | 51.64 | + 5.44 | | 69.59 | + 3.32 | 91.86 | + 3.80 |
| Elav-GAL4/+;UAS*-Alk^WT^*/GAL80^ts^ | 47.67 | + 7.82 | | 67.40 | + 8.11 | 82.95 | + 8.63 |
| Elav-GAL4/+;UAS*-Jeb*/+;GAL80^ts^/+ | 42.60 | + 9.13 | | 66.95 | + 1.84 | 97.61 | + 2.38 |
| Elav-GAL4/UAS*-Alk^CA^*;GAL80^ts^/+ | 60.34 | + 7.65 | | 78.26 | + 8.26 | 85.23 | + 6.27 |
| Elav-GAL4/+;UAS*-Alk^DN^*/GAL80^ts^ | 46.59 | + 5.00 | | ***52.71*** | + ***3.50*** | 92.60 | + 4.73 |
| Elav-GAL4/UAS*-Alk^RNAi^*;GAL80^ts^/+ | 60.91 | + 3.97 | | 79.87 | + 6.06 | 97.80 | + 1.35 |
| **ANOVA** | F_(5,37)_=1.75, P>0.15 | | | F_(5.37)_=3.82, P>0.008 | | F_(5.36)_=1.57, P>0.19 | |
| Alk(38)-GAL4/+;GAL80^ts^/+ | 62.18 | + 4.55 | | 67.89 | + 4.52 | 94.55 | + 2.30 |
| Alk(38)-GAL4/+;UAS*-Alk^WT^*/GAL80^ts^ | 62.02 | + 7.38 | | 61.83 | + 7.87 | 89.89 | + 5.89 |
| Alk(38)-GAL4/UAS*-Jeb*;GAL80^ts^ | 54.73 | + 6.56 | | 59.81 | + 8.46 | 84.21 | + 7.17 |
| UAS*-Alk^CA^*/+;Alk(38)-GAL4/+;GAL80^ts^/+ | 71.57 | + 6.78 | | 60.39 | + 9.81 | 91.31 | + 5.39 |
| Alk(38)-GAL4/+;UAS*-Alk^DN^*/GAL80^ts^ | 74.68 | + 4.44 | | 53.58 | + 5.64 | 93.80 | + 7.21 |
| UAS*-Alk^RNAi^*/+;Alk(38)-GAL4/+;GAL80^ts^ | 73.35 | + 7.43 | | 56.54 | + 7.54 | 87.62 | + 3.23 |
| **ANOVA** | F_(5,37)_=1.74, P>0.15 | | | F_(5,36)_=0.36, P>0.87 | | F_(5,24)_=0.47, P>0.79 | |
| Ras2-GAL4/+;GAL80^ts^/+ | 65.74 | + 3.00 | | 71.58 | + 6.00 | 90.91 | + 3.48 |
| Ras2-GAL4/+;UAS*-Alk^WT^*/GAL80^ts^ | 60.86 | + 9.15 | | 85.80 | + 7.56 | 83.72 | + 6.46 |
| Ras2-GAL4/UAS*-Jeb*;GAL80^ts^/+ | 60.97 | + 2.24 | | 62.66 | + 3.40 | 82.86 | + 2.71 |
| UAS*-Alk^CA^*/+;Ras2-GAL4/+;GAL80^ts^/+ | 61.95 | + 7.32 | | 60.07 | + 6.49 | 83.61 | + 7.02 |
| Ras2-GAL4/+;UAS*-Alk^DN^*/GAL80^ts^ | 65.32 | + 6.45 | | ***44.58*** | ***+ 8.62*** | 82.67 | + 2.64 |
| UAS*-Alk^RNAi^*/+;Ras2-GAL4/+;GAL80^ts^/+ | 64.85 | + 5.73 | | 71.06 | + 3.28 | 87.46 | + 4.65 |
| **ANOVA** | F_(5,36)_=0.20, P>0.95 | | | F_(5,36)_=4.92, P>0.0021 | | F_(5,39)_=0.58, P>0.71 | |
| Alk(38)-GAL4/+;UAS*-Nf1*,E1/E2 | 62.26 | + 4.07 | | 75.95 | 2.78 | 84.34 | + 8.72 |
| Alk(38)-GAL4/+;UAS*-Nf1*/+ | 53.90 | + 2.79 | | 57.46 | 5.03 | 95.00 | + 5.00 |
| Alk(38)-GAL4/+ | 67.19 | + 8.79 | | 74.70 | 4.90 | 90.95 | + 3.46 |
| UAS*-Nf1*,E1/E2 | 62.15 | + 4.60 | | 64.78 | 5.48 | 87.50 | + 5.21 |
| Alk(38)-GAL4;E2 | 58.97 | + 3.84 | | 67.73 | 4.81 | 96.15 | + 3.84 |
| UAS*-Nf1*/+ | 67.39 | + 6.74 | | 58.33 | 7.66 | 86.36 | + 8.70 |
| **ANOVA** | F_(5,38)_=0.85, P>0.52 | | | F_(5,36)_=2.17, P>0.08 | | F_(5,24)_=0.35, P>0.87 | |
| *w^1118^* | 58.73 | + 7.44 | | 49.94 | + 7.44 | 84.26 | + 2.23 |
| *Nf1^E1^* | 50.62 | +5.68 | | 62.26 | + 8.95 | 80.30 | + 3.19 |
| *Nf1^E2^* | 57.07 | + 7.33 | | 44.29 | + 6.90 | 83.43 | + 3.62 |
| *Nf1^E1/E2^* | 59.28 | + 7.90 | | 49.87 | + 2.30 | 84.16 | + 2.33 |
| *Alk^1^*/+ | 68.29 | + 6.35 | | 57.66 | + 6.93 | 85.62 | + 5.44 |
| *Alk^1^*/+;E2 | 67.28 | + 7.00 | | 60.86 | + 3.23 | 87.42 | + 1.36 |
| *Alk^9^*/+ | 52.32 | + 6.77 | | 49.17 | + 6.30 | 81.72 | + 5.09 |
| *Alk^9^*/+;E2 | 45.80 | + 8.65 | | 54.72 | + 8.36 | 85.41 | + 4.12 |
| **ANOVA** | F_(7,57)_=0.57, P>0.77 | | | F_(7,53)_=0.34, P>0.92 | | F_(7,41)_=0.23, P>0.97 | |

**Table S1. Task relevant sensory behaviors.** Avoidance of the aversive odor stimuli (CS) and electric shock (US) is shown for all relevant strains (n>6 for all odor avoidance and n>4 for all shock avoidance experiments). Values in rows indicate the mean Performance Index (PI) + S.E.M. of flies avoiding the corresponding stimulus (Benzaldehyde, Octanol or Electric Shock). ANOVA values are shown below each group tested. Avoidance of strains in each group was compared to that of their proper heterozygous driver control. Values in bold writing indicate statistical significant differences from control strain. All strains were not tested simultaneously, although all strains within a group were. Thus, statistical analyses for performance differences were performed exclusively within each group.
