# Supplementary material for: The Receptor Tyrosine Kinase Alk Controls Neurofibromin Functions in Drosophila Growth and Learning
Source: PLoS Genet. 2011 Sep 15;7(9):e1002281. doi: 10.1371/journal.pgen.1002281 (PMC3174217; doi:10.1371/journal.pgen.1002281)
Supplement: Table S2 — Pupal size alterations result from tissue-specific expression of UAS-dAlk and UAS-Jeb transgenes. The numbers in the ‘Ref.’ column refer to the reference list given below the table. ‘Expression in the 3rd instar larval Nervous System’ briefly describes the main neural tissues in which the Gal4-drivers are expressed. Details come either from already published data or determined by immunohistochemistry we performed on dissected 3rd instar larvae nervous system obtained from crosses of Gal4-drivers to UAS-mCD8::GFP flies. Data on the ‘Effect of UAS-dAlk transgenes on pupal size’ were obtained from crosses of Gal4-drivers to UAS-dAlkWT, UAS-Jeb, UAS-dAlkCA, UAS-dAlkDN, UAS-dAlkRNAi strains. Each cross was set up using an equal number of females per vial and at least 15 pupae of both sexes were measured for each Gal4-driver (n = 15–50). Pupal size measurements were performed as described in the ‘Materials and Methods’ section. The resulting effect on pupal size is qualitatively represented by upward (↑ for increase), or downward (↓ for reduced) arrows for those Gal4-drivers that significantly altered pupal size, and by horizontal arrrows (→) for those that do not significantly alter size (Dunnett's test, p<0.001). Crosses (†) represent larval lethality, and thus not measurable at the pupal stage. Abbreviations: AL: Antenna lobe; CB: central brain; CCAP: Crustacean cardioactive peptide; CNS: central nervous system; DPM: Dorsal paired medial neurons; MB: Mushroom Body neurons; MBextr: extrinsic mushroom bodies neurons (presynaptic neurons that project to MBs); MBintr: intrinsic mushroom bodies neurons (postsynaptic neurons constituent of mushroom bodies); PN; Projection neurons; RG: ring gland; SG: salivary glands; VG: ventral ganglion. (DOC) [file pgen.1002281.s007.doc]

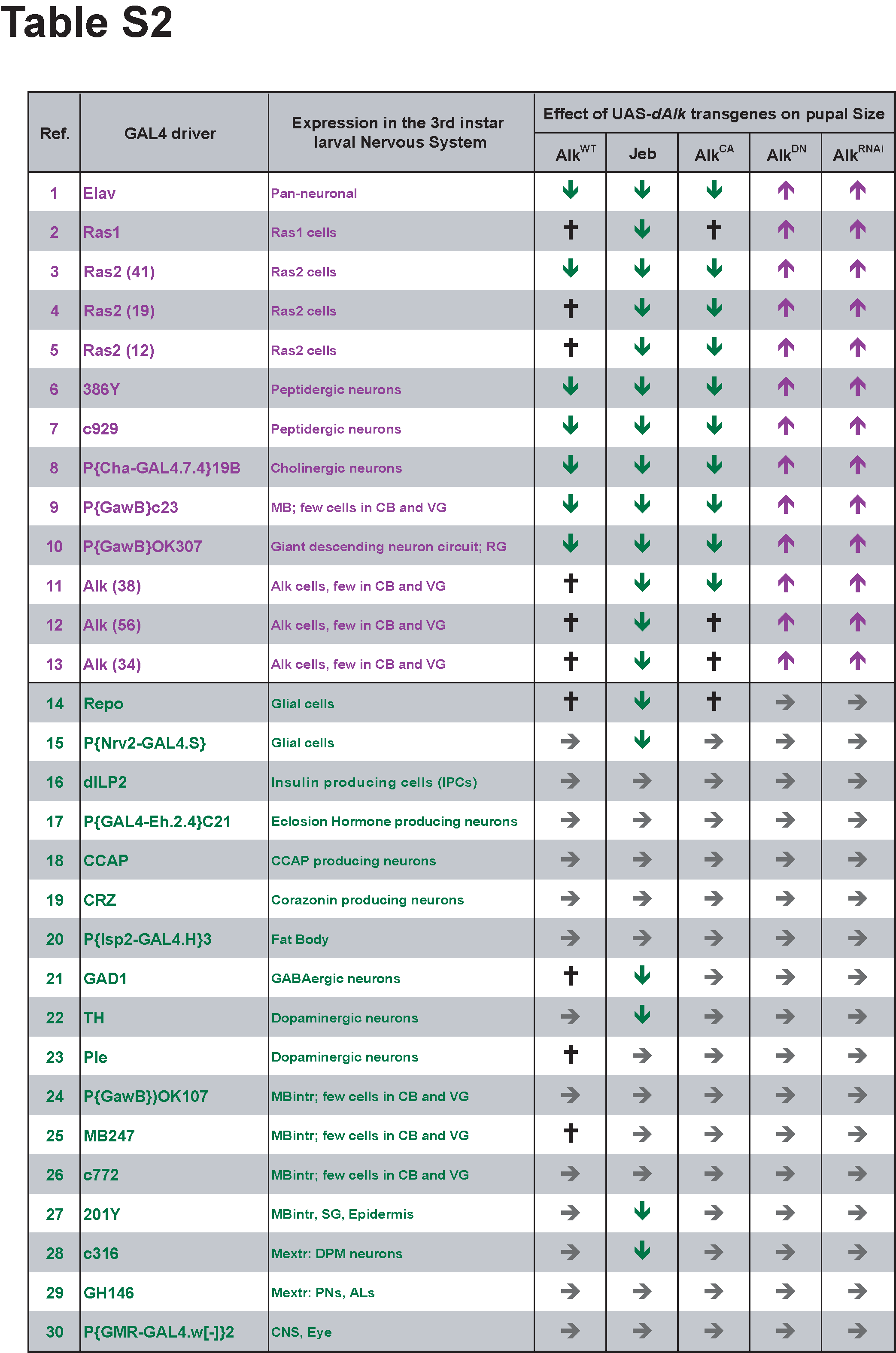


**Origin of Gal4 drivers.**

1. Lin DM, and Goodman CS (1994) Ectopic and increased expression of Fasciclin II alters motoneuron growth cone guidance. Neuron 13: 507- 523.

*Provided by Bloomington Drosophila Stock Center (B458).*

2-5. Walker JA, Tchoudakova AV, McKenney PT, Brill S, Wu D, Cowley GS, Hariharan IK, Bernards A (2006) Reduced growth of Drosophila neurofibromatosis 1 mutants reflects a non-cell-autonomous requirement for GTPase-Activating Protein activity in larval neurons. Genes Dev. 20:3311-23.

6. Taghert PH, Hewes RS, Park JH, O'Brien MA, Han M, Peck ME (2001) Multiple amidated neuropeptides are required for normal circadian locomotor rhythms in Drosophila. J Neurosci 21: 6673-6686.

*Provided by Dr. P.H. Taghert.*

7. Hewes RS, Schaefer AM, Taghert PH (2000) The cryptocephal gene (ATF4) encodes multiple basic-leucine zipper proteins controlling molting and metamorphosis in Drosophila. Genetics 155: 1711-1723.

*Provided by Dr. P.H. Taghert.*

8. Salvaterra PM, and Kitamoto T (2001) Drosophila cholinergic neurons and processes visualized with Gal4/UAS-GFP. Brain Res. Gene Expression Patterns 1: 73-82.

*Provided by Bloomington Drosophila Stock Center (B6798).*

9. *Provided by Bloomington Drosophila Stock Center (B2703).*

10. *Provided by Bloomington Drosophila Stock Center (B6488).*

11-13. This study.

14. Sepp KJ, Schulte J, Auld VJ (2001) Peripheral glia direct axon guidance across the CNS/PNS transition zone. Dev Biol 238: 47-63.

15. *Provided by Bloomington Drosophila Stock Center (B6800).*

16. Rulifson EJ, Kim SK, Nusse R (2002) Ablation of insulin-producing neurons in flies: growth and diabetic phenotypes. Science 296: 1118-1120.

*Provided by Dr. E.J. Rulifson.*

17. McNabb SL, Baker JD, Agapite J, Steller H, Riddiford LM, Truman JW (1997) Disruption of a behavioral sequence by targeted death of peptidergic neurons in Drosophila. Neuron 19: 813-823.

*Provided by Bloomington Drosophila Stock Center (B6301).*

18. Park JH, Schroeder AJ, Helfrich-Forster C, Jackson FR, Ewer J (2003) Targeted ablation of CCAP neuropeptide-containing neurons of Drosophila causes specific defects in execution and circadian timing of ecdysis behavior. Development 130: 2645–2656.

19. Choi YJ, Lee G, Hall JC, Park JH (2005) Comparative analysis of Corazonin encoding genes (Crz's) in Drosophila species and functional insights into Crz expressing neurons. J Comp Neurol 482: 372–385.

*Provided by Dr. J.H. Park.*

20. *Provided by Bloomington Drosophila Stock Center (B6357).*

21. Ng M, Roorda RD, Lima SQ, Zemelman BV, Morcillo P, Miesenböck G (2002) Transmission of olfactory information between three populations of neurons in the antennal lobe of the fly. Neuron 36: 463-474.

22. Friggi-Grelin F, Coulom H, Meller M, Gomez D, Hirsh J, Birman S (2003) Targeted gene expression in Drosophila dopaminergic cells using regulatory sequences from tyrosine hydroxylase. J Neurobiol 54: 618-627.

23. *Provided by Bloomington Drosophila Stock Center (B8848).*

24. Lee T, Lee A, Luo L (1999) Development of the Drosophila mushroom bodies: sequential generation of three distinct types of neurons from a neuroblast. Development 126: 4065-4076.

*Provided by Bloomington Drosophila Stock Center (B854).*

25. Zars T, Fischer M, Schulz R, Heisenberg M (2000) Localization of a short-term memory in Drosophila. Science 288: 672–675.

26-27. Yang MY, Armstrong JD, Vilinsky I, Strausfeld NJ, Kaiser K (1995) Subdivision of the Drosophila mushroom bodies by enhancer-trap expression patterns. Neuron 15: 45-54.

*Provided by Dr. J.D. Armstrong.*

28. Waddell S, Armstrong JD, Kitamoto T, Kaiser K, Quinn WG (2000) The amnesiac gene product is expressed in two neurons in the Drosophila brain that are critical for memory. Cell 103: 805-813.

*Provided by Dr. S. Waddell.*

29. Stocker RF, Heimbeck G, Gendre N, de Belle JS (1997) Neuroblast ablation in Drosophila P[Gal4] lines reveals origins of olfactory interneurons. J Neurobiol. 32: 443-456.

30. Freeman M (1996) Reiterative use of the EGF receptor triggers differentiation of all cell types in the Drosophila eye. Cell 87: 651–660.

*Provided by Bloomington Drosophila Stock Center (B9146).*
