# Supplementary material for: The Receptor Tyrosine Kinase Alk Controls Neurofibromin Functions in Drosophila Growth and Learning
Source: PLoS Genet. 2011 Sep 15;7(9):e1002281. doi: 10.1371/journal.pgen.1002281 (PMC3174217; doi:10.1371/journal.pgen.1002281)
Supplement: Table S3 — Results of planned comparisons for Figure 2, Figure 3, Figure 6, and Figure 7 in the main article. The scores of all genotypes were compared per group (each experimental group separated by an empty row) with the relevant genotype listed first (indicated by #). Significant differences are denoted by the star sign. The level of significance was adjusted for the experimentwise error rate. (DOC) [file pgen.1002281.s008.doc]

| **Figure 2A** | **Genotype** | **Mean + SEM** | **t-Ratio** | **P Value** |
| --- | --- | --- | --- | --- |
|  | ElavGAL4/+;Gal80^ts^/+ | 75.70 + 1.82 |  |  |
|  | ElavGAL4/+;UAS-ALK^WT^/Gal80^ts^ | 54.11 + 2.36 | 6.08 | <0.0001* |
|  | ElavGAL4/+;UAS-Jeb/+;Gal80^ts^/+ | 60.81 + 1.06 | 4.19 | 0.0002* |
|  | ElavGAL4/UAS-ALK^CA^;Gal80^ts^/+ | 64.17 + 5.52 | 3.25 | 0.00025* |
|  | ElavGAL4/+;UAS-ALK^DN^/Gal80^ts^ | 82.43 + 1.12 | -1.98 | 0.027 |
|  | ElavGAL4/UAS-ALK^RNAi^;Gal80^ts^/+ | 86.42 + 2.03 | -3.15 | 0.0016* |
|  |  |  |  |  |
| **Figure 2B** | RAS2GAL4/+; Gal80^ts^/+ | 75.19 + 0.94 |  |  |
|  | RAS2GAL4/+;UAS-ALK^WT^/ Gal80^ts^ | 58.69 + 5.80 | 3.37 | 0.0017* |
|  | RAS2GAL4/UAS-Jeb; Gal80^ts^/+ | 54.75 + 6.33 | 4.36 | <0.0001* |
|  | UAS-ALK^CA^/+;RAS2GAL4/+; Gal80^ts^/+ | 60.19 + 2.77 | 3.06 | 0.0039* |
|  | RAS2GAL4/+;UAS-ALK^DN^/ Gal80^ts^ | 84.91 + 1.25 | -2.26 | 0.01* |
|  | UAS-ALK^RNAi^/+;RAS2GAL4/+; Gal80^ts^/+ | 83.99 + 1.10 | -1.94 | 0.029 |
|  |  |  |  |  |
| **Figure 2C** | ALK(38)GAL4/+;Gal80^ts^/+ | 71.86 + 3.49 |  |  |
|  | ALK(38)GAL4/+;UAS-ALK^WT^/Gal80^ts^ | 49.60 + 6.80 | 3.60 | 0.0011* |
|  | ALK(38)GAL4/UAS-Jeb;Gal80^ts^ | 54.09 + 3.65 | 2.87 | 0.0074* |
|  | UAS-ALK^CA^/+;ALK(38)GAL4/+; Gal80^ts^/+ | 51.77 + 5.08 | 3.25 | 0.0028* |
|  | ALK(38)GAL4/+;UAS-ALK^DN^/Gal80^ts^ | 73.85 + 2.05 | -0.32 | 0.37 |
|  | UAS-ALK^RNAi^/+;ALK(38)GAL4/+; Gal80^ts^ | 73.17 + 3.57 | -0.21 | 0.41 |

|  | **Genotype** | **Mean + SEM** | **t-Ratio** | **P value** |
| --- | --- | --- | --- | --- |
| **Figure 3A** | ElavGAL4/+ | 2993 + 90 |  |  |
|  | ElavGAL4/+;UAS-ALK^WT^/+ | 2579 + 33 | 11.76 | <0.0001* |
|  | ElavGAL4/+;UAS-Jeb/+ | 2569 + 18 | 12.04 | <0.0001* |
|  | ElavGAL4/UAS-ALK^CA^ | 2732 + 31 | 7.33 | <0.0001* |
|  | ElavGAL4/+;UAS-ALK^DN^/+ | 3482 + 16 | -13.33 | <0.0001* |
|  | ElavGAL4/UAS-ALK^RNAi^ | 3283 + 26 | -8.01 | <0.0001* |
|  |  |  |  |  |
| **Figure 3B** | RAS2GAL4/+ | 3013 + 24 |  |  |
|  | RAS2GAL4/+;UAS-ALK^WT^/+ | 2397 + 24 | 18.00 | <0.0001* |
|  | RAS2GAL4/UAS-Jeb | 2380 + 24 | 18.50 | <0.0001* |
|  | UAS-ALK^CA^/+;RAS2GAL4/+ | 2620 + 22 | 11.49 | <0.0001* |
|  | RAS2GAL4/+;UAS-ALK^DN^/+ | 3624 + 15 | -17.85 | <0.0001* |
|  | UAS-ALK^RNAi^/+;RAS2GAL4/+ | 3358 + 30 | -10.09 | <0.0001* |
|  |  |  |  |  |
| **Figure 3C** | ALK(38)GAL4/+ | 2999 + 20 |  |  |
|  | ALK(38)GAL4/+;UAS-ALK^WT^/+ | - | - | - |
|  | ALK(38)GAL4/UAS-Jeb | 2530 + 20 | 15.15 | <0.0001* |
|  | UAS-ALK^CA^/+;ALK(38)GAL4/+ | 2334 + 32 | 21.45 | <0.0001* |
|  | ALK(38)GAL4/+;UAS-ALK^DN^/+ | 3354 + 16 | -11.47 | <0.0001* |
|  | UAS-ALK^RNAi^/+;ALK(38)GAL4/+ | 3208 + 12 | -6.73 | <0.0001* |
|  |  |  |  |  |
| **Figure 3D** | 386YGAL4/+ | 2994 + 32 |  |  |
|  | 386YGAL4/+;UAS-ALK^WT^/+ | 2547 + 24 | 10.09 | <0.0001* |
|  | 386YGAL4/UAS-Jeb | 2267 + 19 | 16.42 | <0.0001* |
|  | UAS-ALK^CA^/+;386YGAL4/+ | 2648 + 32 | 7.80 | <0.0001* |
|  | 386YGAL4/+;UAS-ALK^DN^/+ | 3522 + 49 | -11.37 | <0.0001* |
|  | UAS-ALK^RNAi^/+;386YGAL4/+ | 3267 + 26 | -6.32 | <0.0001* |

|  | **Genotype** | **Mean + SEM** | **t-Ratio** | **P Value** |
| --- | --- | --- | --- | --- |
| **Figure 6A** | ALK(38)GAL4/+;UAS-NF1,E1/E2 | 3199 + 18 |  |  |
|  | ALK(38)GAL4/+;UAS-NF1/+ | 3307 + 11 | -4.06 | <0.0001* |
|  | ALK(38)GAL4/+ | 3247 + 22 | 1.63 | 0.10 |
|  | UAS-NF1,E1/E2 | 2624 + 26 | 19.75 | <0.0001* |
|  | ALK(38)GAL4;E2 | 2642 + 33 | 17.06 | <0.0001* |
|  | | | | |
| **Figure 6B** | E2 | 2594 + 11 |  |  |
|  | W^1118^ | 3220 + 13 | 33.87 | <0.0001* |
|  | ALK^1^/+ | 3201 + 14 | -35.07 | <0.0001* |
|  | ALK^1^/+;E2 | 2857 + 22 | -11.98 | <0.0001* |
|  | ALK^9^/+ | 3256 + 11 | 35.85 | <0.0001* |
|  | ALK^9^/+;E2 | 2923 + 20 | 12.93 | <0.0001* |
|  | | | | |
| **Figure 6C** | NO VEHICLE | 2661 + 10 |  |  |
|  | 0 nM | 2650 + 5 | 0.99 | 0.32 |
|  | 100 nM | 2721 + 9 | 4.79 | <0.0001* |
|  | 1 uM | 2789 + 6 | 10.83 | <0.0001* |
|  | 10 uM | 2786 + 9 | 9.69 | <0.0001* |
|  | 100 uM | 2641 + 9 | 1.57 | 0.11 |
|  | | | | |
| **Figure 6D** | ELAVGAL4/+;E2 | 2644 + 17 |  |  |
|  | ELAVGAL4/+;E2/UAS-ALK^EC^,E2 | 3110 + 15 | 19.13 | <0.0001* |
|  | ELAVGAL4/UAS-ALK^RNAi^;E2 | 3001 + 18 | 14.76 | <0.0001* |
|  | ELAVGAL4/+ | 3145 + 16 | 20.58 | <0.0001* |
|  |  |  |  |  |
|  | 386YGAL4/+;E2 | 2487 + 13 |  |  |
|  | 386YGAL4/+;E2/UAS-ALK^EC^,E2 | 2952 + 11 | 26.30 | <0.0001* |
|  | UAS-ALK^RNAi^/+;386YGAL4/+;E2 | 2891 + 11 | 23.79 | <0.0001* |
|  | 386YGAL4/+ | 3089 + 17 | 29.15 | <0.0001* |
|  |  |  |  |  |
|  | RAS2GAL4/+;E2 | 2646 + 23 |  |  |
|  | RAS2GAL4/+;E2/UAS-ALK^EC^,E2 | 3017 + 20 | 14.20 | <0.0001* |
|  | UAS-ALK^RNAi^/+;RAS2GAL4/+;E2 | 2956 + 18 | 11.22 | <0.0001* |
|  | RAS2GAL4/+ | 3212 + 9 | 24.86 | <0.0001* |
|  |  |  |  |  |
|  | E2;OK107GAL4/+ | 2617 + 14 |  |  |
|  | E2/UAS-ALK^EC^,E2;OK107GAL4/+ | 2610 + 14 | 0.36 | 0.71 |
|  | UAS-ALK^RNAi^/+;E2;OK107GAL4/+ | 2624 + 16 | 0.37 | 0.71 |
|  | OK107/+ | 3206 + 10 | 30.66 | <0.0001* |
|  |  |  |  |  |
|  | CCAPGAL4/+;E2 | 2697 + 18 |  |  |
|  | CCAPGAL4/+;E2/UAS-ALK^EC^,E2 | 2706 + 21 | 0.32 | 0.72 |
|  | UAS-ALK^RNAi^/+;CCAPGAL4/+;E2 | 2736 + 15 | 1.53 | 0.12 |
|  | CCAPGAL4/+ | 3295 + 17 | 22.61 | <0.0001* |

|  | **Genotype** | **Mean + SEM** | **t-Ratio** | **P Value** |
| --- | --- | --- | --- | --- |
| **Figure 7A** | Alk(38)-GAL4/+;UAS*-Nf1*,E1/E2 | 80.70 + 1.79 |  | # |
|  | Alk(38-)-GAL4/+;UAS*-Nf1*/+ | 77.19 + 0.54 | 0.55 | 0.58 |
|  | Alk(38)-GAL4/+ | 81.98 + 4.10 | 0.24 | 0.81 |
|  | UAS*-Nf1*,E1/E2 | 45.51 + 3.97 | 7.65 | <0.0001* |
|  | Alk(38)-GAL4;E2 | 50.19 + 5.45 | 6.39 | <0.0001* |
|  | UAS*-Nf1*/+ | 76.12 + 3.20 | 0.96 | 0.34 |
|  | | | | |
| **Figure 7B** | E2 | 44.95 + 3.01 |  | # |
|  | *w^1118^* | 71.69 + 1.57 | -6.12 | <0.0001* |
|  | *Alk^1^*/+ | 63.07 + 4.14 | -3.88 | 0.0002* |
|  | *Alk^1^*/+;E2 | 57.05 + 3.06 | -2.86 | 0.0054* |
|  | *Alk^9^*/+ | 66.51 + 3.72 | -4.73 | <0.0001* |
|  | *Alk^9^*/+;E2 | 59.41 + 3.98 | -3.10 | 0.0027* |
|  | | | | |
| **Figure 7C** | E2 0 nM | 46.52 + 1.44 |  | # |
|  | E2 10 nM | 55.90 + 2.32 | -3.34 | 0.0013* |
|  | E2 100 nM | 60.47 + 1.66 | -5.22 | <0.0001* |
|  | E2 1 mM | 51.85 + 2.47 | -1.95 | 0.054 |
|  | E2 100 mM | 53.27 + 2.49 | -2.40 | 0.018 |
|  | *w^1118^* No Vehicle | 67.27 + 1.95 | -7.93 | <0.0001* |
|  | *w^1118^* 0 nM | 68.30 + 1.45 | -8.32 | <0.0001* |
|  | | | | |
| **Figure 7D** | *UNINDUCED* | | | |
|  | Elav-GAL4/+;G80^ts^/+ | 80.64 + 1.40 |  | # |
|  | Elav-GAL4/+;E2,G80^ts^/E2 | 57.60 + 7.11 | 2.90 | 0.0058* |
|  | Elav-GAL4/+;E2,G80^ts^/UAS*-Alk^DN^*,E2 | 56.63 + 5.91 | 3.03 | 0.0042* |
|  | Elav-GAL4/UAS*-Alk^RNAi^*;E2,G80^ts^/E2 | 59.96 + 4.67 | 2.81 | 0.0073* |
|  | Elav-GAL4/+;E1,G80^ts^/E2 | 54.27 + 5.55 | 3.47 | 0.0012* |
|  | Elav-GAL4/+;E2,G80^ts^/UAS*-Nf1*,E1 | 55.23 + 6.85 | 3.34 | 0.0017* |
|  | Elav-GAL4/UAS*-Alk^RNAi^*;E1,G80^ts^/E2 | 48.74 + 6.67 | 4.20 | <0.0001* |
|  | *INDUCED* | | | |
|  | Elav-GAL4/+;G80^ts^/+ | 76.55 + 3.09 |  | # |
|  | Elav-GAL4/+;E2,G80^ts^/E2 | 52.05 + 3.62 | 3.78 | 0.0004* |
|  | Elav-GAL4/+;E2,G80^ts^/UAS*-Alk^DN^*,E2 | 74.27 + 4.79 | 0.35 | 0.72 |
|  | Elav-GAL4/UAS*-Alk^RNAi^*;E2,G80^ts^/E2 | 71.07 + 5.36 | 0.87 | 0.38 |
|  | Elav-GAL4/+;E1,G80^ts^/E2 | 44.76 + 5.05 | 5.23 | <0.0001* |
|  | Elav-GAL4/+;E2,G80^ts^/UAS-*Nf1*,E1 | 78.99 + 3.97 | 0.40 | 0.68 |
|  | Elav-GAL4/UAS*-Alk^RNAi^*;E1,G80^ts^/E2 | 73.61 + 4.22 | 0.47 | 0.64 |
|  |  |  |  |  |
|  | Ras2-GAL4/+;E2,G80^ts^/E2 | 40.41 + 3.19 |  | # |
|  | Ras2-GAL4/+;G80^ts^/+ | 70.74 + 1.33 | -7.56 | <0.0001* |
|  | Ras2-GAL4/+;E2,G80^ts^/UAS*-Alk^DN^*,E2 | 61.12 + 3.66 | -4.15 | 0.0002* |
|  | UAS*-Alk^RNAi^*/+;Ras2-GAL4/+;E2,G80^ts^/E2 | 63.51 + 5.11 | -5.18 | <0.0001* |
|  | Ras2-GAL4/+;E2,G80^ts^/UAS*-Nf1*,E1 | 57.90 + 2.59 | -3.93 | 0.0004* |
|  |  |  |  |  |
|  | Alk(38)-GAL4/+;E2,G80^ts^/E2 | 56.02 + 5.22 |  | # |
|  | Alk(38)-GAL4/+;G80^ts^/+ | 77.53 + 2.50 | -3.35 | 0.002* |
|  | Alk(38)-GAL4/+;E2,G80^ts^/UAS*-Alk^DN^*,E2 | 81.81 + 6.37 | -3.66 | 0.0009* |
|  | UAS*-Alk^RNAi^*/+;Alk(38)-GAL4/+;E2,G80^ts^/E2 | 75.09 + 4.37 | -2.89 | 0.0066* |
|  | Alk(38)-GAL4/+;E2,G80^ts^/UAS*-Nf1*,E1 | 76.11 + 4.30 | -3.12 | 0.0037* |
|  |  |  |  |  |
|  | c772-GAL4 /+;E2,G80^ts^/E2 | 37.48 + 9.51 |  | # |
|  | c772-GAL4/+;G80^ts^/+ | 66.96 + 2.02 | -3.62 | 0.0009* |
|  | C772-GAL4/+;E2,G80^ts^/UAS*-Alk^DN^*,E2 | 42.66 + 5.91 | -0.59 | 0.55 |
|  | UAS*-Alk^RNAi^*/+;c772-GAL4/+;E2,G80^ts^/E2 | 33.95 + 5.98 | 0.39 | 0.69 |
|  | c772-GAL4/+;E2,G80^ts^/UAS*-Nf1*,E1 | 32.74 + 6.20 | 0.54 | 0.58 |

**Table S3. Results of planned comparisons for Figure 2, Figure 3, Figure 6 and Figure 7 in the main article.** The scores of all genotypes were compared per group (each experimental group separated by an empty row) with the relevant genotype listed first (indicated by #). Significant differences are denoted by the star sign. The level of significance was adjusted for the experimentwise error rate.
